# Conserved sex-biased DNA methylation patterns target key developmental genes and reproductive function in two Poeciliidae species

**DOI:** 10.1093/g3journal/jkag228

**Published:** 2026-08-19

**Authors:** David C H Metzger, Judith E Mank

**Affiliations:** Department of Zoology and Biodiversity Research Centre, The University of British Columbia, Vancouver, BC, Canada V6T 1Z4; Department of Zoology and Biodiversity Research Centre, The University of British Columbia, Vancouver, BC, Canada V6T 1Z4

**Keywords:** epigenetics, comparative epigenomics, *Poecilia reticulata*, *Poecilia wingei*, whole-genome bisulfite sequencing

## Abstract

Sexual dimorphism provides a powerful framework for studying how regulatory mechanisms generate phenotypic divergence from a shared genome. Many sex-specific traits arise from differences in gene expression, which can be mediated by epigenetic mechanisms such as DNA methylation. These mechanisms contribute to the organization of gene regulatory variation across tissues, genes, and chromosomes, with potential consequences for the emergence and maintenance of sexually dimorphic phenotypes. Using a comparative epigenomic approach we investigate sex-biased DNA methylation in gonad and muscle tissue across 2 closely related Poeciliidae species, *Poecilia reticulata* and *Poecilia wingei*. We address 3 questions: (i) how conserved are sex-biased DNA methylation patterns across species and tissues; (ii) are conserved sex-biased methylation patterns associated with genes involved in developmental and transcriptional regulation; and (iii) are sex-biased epigenetic patterns enriched on the sex chromosome? We identify extensive conservation of sex-biased differentially methylated regions in gonadal tissue, with strong cross-species concordance, whereas muscle shows little conservation of sex-specific differential methylation. Conserved male-hypomethylated regions are enriched near genes involved in developmental transcriptional regulation and gonadal differentiation. Methylation–expression coupling distinguishes sex-biased reproductive gene functions, with male-biased genes enriched for spermatogenesis and motility and female-biased genes enriched for sperm–egg interaction processes. Sex-biased DNA methylation is broadly distributed across the genome, with localized regulatory regions on the sex chromosome. Together, these results show that conserved sex-biased DNA methylation is highly tissue-specific and associated with reproductive regulatory function, supporting a model in which gonadal epigenetic architecture reflects conserved regulatory organization underlying sexual dimorphism.

## Introduction

Males and females share largely the same genome, yet often exhibit substantial differences in morphology, physiology, and behavior, underscoring the role of regulatory mechanisms in generating phenotypic diversity. Epigenetic marks such as DNA methylation are dynamic biochemical mechanisms that can influence gene expression and have the potential to contribute to this diversity by shaping regulatory variation without requiring changes to the underlying DNA sequence (Jaenisch and Bird 2003; Jones 2012). At a mechanistic level, hypomethylation is typically associated with transcriptional activation, whereas hypermethylation is associated with transcriptional repression. However, these relationships are context dependent, and DNA methylation can have distinct or even opposing associations with gene expression depending on genomic location, gene class, and developmental context (Jones 2012). Moreover, because DNA methylation is environmentally responsive and subject to developmental and transgenerational reprogramming, distinguishing evolutionarily conserved, functionally constrained regulatory variation depends on comparative approaches that can isolate stable patterns from transient, context-dependent epigenetic states (Heard and Martienssen 2014).

Distinguishing functionally constrained regulatory variation from environmentally or developmentally labile epigenetic states remains a central challenge in comparative epigenomics. Because DNA methylation patterns can be reset across generations and are sensitive to environmental conditions, not all observed variation is expected to have functional or evolutionary significance (Ortega-Recalde and Hore 2019). Conserved methylation patterns identified through comparative analyses across species are more likely to reflect regulatory elements under stabilizing selection than transient or context-dependent variation, although conservation alone does not establish that these elements are functionally active in the tissue or developmental stage being examined (Long et al. 2013; Lowdon et al. 2016; Zhou et al. 2017). Identifying such conserved features enables the resolution of functionally constrained regulatory variation from environmentally or developmentally labile epigenetic states.

Within this framework, sexual dimorphism provides a particularly useful system to investigate these questions. Many sex-specific traits arise from differences in gene expression achieved through differential regulation (Mank 2017). Consistent with this, sex-biased DNA methylation has been documented across a wide range of vertebrate taxa and is associated with differences in gene expression between males and females (Waters et al. 2018; Zhou et al. 2019; Bain et al. 2021). Epigenetic mechanisms, including DNA methylation, are therefore likely to contribute to the regulatory architecture underlying these differences. Testing this prediction requires a comparative framework that integrates multiple tissues and species, allowing conserved patterns to be distinguished from lineage-specific or context-dependent variation.

Within the genome, the sex chromosomes provide a key framework for understanding the evolution of sex-biased regulatory architecture. Sex chromosomes form the primary genomic basis of genetic sex determination in many species and are therefore integrated into sex-specific gene regulatory networks. Theory predicts that sex chromosomes can act as focal points for the accumulation of sex-biased regulatory variation (Rice 1984; Lenormand et al. 2020). These theoretical predictions suggest that sex-biased regulatory variation may also be reflected at the level of epigenetic regulation, including DNA methylation, which contributes to sex-specific regulatory variation. Although highly derived, heteromorphic sex chromosomes often exhibit pronounced sex-biased epigenetic features (Metzger and Schulte 2018; Muyle et al. 2021; Metzger et al. 2023), it is not clear whether these patterns arise as a consequence of long-term chromosomal differentiation or reflect more general features of sex-specific regulatory architecture. Organisms with young or homomorphic sex chromosomes provide an opportunity to address this question by separating sex-biased regulatory evolution from the consequences of long-term sex chromosome divergence.

The Trinidadian guppy (*Poecilia reticulata*) and its sister species Endler's guppy (*Poecilia wingei*) (Pollux et al. 2014) provide a system in which sexually dimorphic phenotypes can be examined. Both species exhibit pronounced sexual dimorphism in size, coloration, and behavior (Poeser et al. 2005), and their close evolutionary relationship enables identification of methylation patterns that are conserved across species, providing a robust framework for distinguishing functionally constrained epigenetic variation from more labile patterns. Furthermore, they share relatively undifferentiated sex chromosomes estimated to have originated approximately 20 million years ago (Darolti et al. 2023, 2019; Fong et al. 2023; Tripathi et al. 2009). Despite localized divergence, there is little evidence of widespread Y chromosome degeneration (Darolti et al. 2020; van der Bijl et al. 2025). As a result, sexually dimorphic phenotypes in these species are likely to be driven primarily by regulatory mechanisms rather than changes in underlying genetic sequence.

In this study, we use a comparative epigenomic approach to investigate the evolution and organization of sex-biased DNA methylation across tissues and species. Specifically, we address 3 key questions. First, how evolutionarily conserved are sex-biased DNA methylation patterns, and are they concentrated in specific tissues? Second, are conserved sex-biased methylation patterns associated with genes involved in developmental and transcriptional regulatory processes? Third, are sex-biased epigenetic patterns preferentially associated with the sex chromosome in a system with largely homomorphic sex chromosomes, or are they broadly distributed across the genome? To address these questions, we generated whole-genome bisulfite sequencing (WGBS) data from gonad and muscle tissue of both sexes in *P. reticulata* and *P. wingei*, and integrated these data with RNA-seq from the same tissues. This comparative framework allows us to identify conserved, tissue-specific patterns of sex-biased DNA methylation and to assess their relationship with gene expression and genomic context.

## Materials and methods

### Sample collection and WGBS

Adult male and virgin females were collected from laboratory populations of *P. reticulata* and *P. wingei*. The *P. reticulata* lab strain is an outbred laboratory population originating from a high predation population in the Quare River, Trinidad (Kotrschal et al. 2013). The *P. wingei* laboratory population was obtained from a UK fish hobbyist. All fish were maintained under a standard diet of fancy guppy flake and artemia, at 25 °C and a 12h–12 h light cycle. Following a lethal dose of MS-222, we immediately dissected muscle and gonad tissue, which were then stored in 95% ethanol for subsequent DNA isolation. Muscle tissue was collected from the caudal region, extending from the anal pore to the base of the tail, with skin and bone removed prior to processing. This region was selected to obtain sufficient skeletal muscle tissue for WGBS while maintaining consistent sampling across individuals.

Interindividual DNA methylation variation can be attributed to genetic differences as well as environmental factors and life experiences (Jaenisch and Bird 2003). To reduce the effects of interindividual variation, we pooled tissue from 5 individuals for each sequencing library. While this approach provides an integrated estimate of methylation patterns within each biological group, it also limits the ability to assess individual-level variability or heterogeneous methylation states. Future studies using individually resolved methylomes will be valuable for quantifying within-population variation in DNA methylation. Five libraries were created for each tissue for both species. A total of 25 male and 25 female fish were sampled for each species. The same individuals that were pooled for the gonad samples were also pooled for muscle. We stored tissue at 4 °C for 24 to 72 h prior to extracting DNA with a DNAeasy Blood and Tissue Kit (Qiagen) with on-column RNase A treatment following the manufacturer's protocol. Resulting genomic DNA samples were frozen at −20 °C, and then shipped overnight on dry ice to Génome Québec for bisulfite conversion, library preparation, and sequencing on the Illumina HiSeqX PE150 platform.

### DNA methylation analysis

Raw paired-end WGBS reads were quality trimmed using Trimmomatic v0.39 (Bolger et al 2014) with paired-end adapter removal (TruSeq3), a sliding-window quality filter (4 bp window, minimum mean quality 15), and a minimum read length of 36 bp. Trimmed reads from both species were aligned to the *P. reticulata* reference genome (GCF_000633615.1) using BSBolt (Farrell et al 2021). Duplicate-marked BAM files were produced using SAMtools fixmate, sort, and markdup (Li et al 2009), and per-CpG methylation was extracted using the BSBolt CallMethylation module with no minimum coverage filter applied at the calling stage. Average mapping efficiency was similar across species, sexes, and tissues: in *P. reticulata*, 96.79% (ovary), 97.32% (female muscle), 96.79% (testis), and 96.55% (male muscle); and in *P. wingei*, 96.16% (ovary), 96.21% (female muscle), 96.22% (testis), and 96.11% (male muscle). Sequencing and alignment quality metrics, including read counts, mapping efficiencies, duplicate rates, and CpG coverage statistics for all WGBS libraries, are provided in Supplementary Table 1. To assess sex-specific coverage patterns across Chromosome 12, genomic coverage was calculated for male and female samples within 25 kb nonoverlapping genomic windows. Mean coverage was calculated separately for each sex within each window and normalized relative to genome-wide mean coverage to account for differences in sequencing depth among samples. Male:female coverage ratios were calculated as the normalized male coverage divided by the normalized female coverage for each window, followed by log_2_ transformation [log_2_(M:F)]. Differences between species were calculated by subtracting the log_2_(M:F) values of *P. wingei* from *P. reticulata* within corresponding genomic windows.

Bisulfite conversion efficiency was estimated for each sample from genome-wide non-CpG (CHG and CHH context) methylation levels. Across all 39 samples, mean conversion efficiency was 99.25 ± 0.16% (Supplementary Table 2). For genome-wide methylation summaries and principal component analysis (PCA) analyses, CpG sites were filtered to retain loci with ≥10× coverage in every sample to ensure reliable site-level methylation estimates for analyses that do not model coverage-associated uncertainty. Of the 24,798,899 CpGs observed across all samples, 122,409 (0.5%) met this stringent criterion and were retained for these analyses. The low retention reflects the requirement for sufficient coverage across all biological replicates simultaneously. Differential methylation analyses were performed separately using the complete set of 24,798,899 CpGs as input. Methylation levels are reported as the proportion of methylated cytosines at each CpG site and were summarized across the genome to obtain per-sample mean methylation estimates. To visualize overall patterns of methylation variation among samples, PCA was performed on the complete matrix of per-CpG methylation values. Prior to PCA, CpG sites with zero variance across samples and sites containing nonfinite values were removed. The remaining sites were centered and scaled before running PCA using the prcomp function in R (R Core Team).

A de novo CpGi annotation was compiled for the *P. reticulata* reference genome by combining 2 complementary approaches. Hypomethylated regions (HMRs) were identified in testis, ovary, and muscle tissues using MethPipe (Song et al 2013), and CpG-rich regions were independently predicted using makeCGI (Wu et al 2010), which identifies CpG islands based on a hidden Markov model (HMM) trained on CpG dinucleotide composition. The resulting HMR and HMM-based predictions were combined to produce a unified de novo CpGi annotation. To characterize the sequence composition of CpG islands relative to other genomic features, CpG observed-to-expected ratios (CpG O/E) and GC content were calculated for CpGi sequences and compared against HMRs identified in each tissue type. CpG O/E distributions were processed to remove zero values and cap extreme values at a maximum of 10, and outliers were trimmed using an adaptive IQR-based method in which the most conservative multiplier (ranging from 2× to 5× IQR) that excluded no more than 1% of observations was applied. To characterize methylation within CpG islands specifically, per-CpG methylation values from the genome-wide matrix were intersected with CpGi coordinates using the findOverlaps function from the GenomicRanges package in R, and mean methylation per island was calculated for each sample. Island-level methylation distributions were visualized separately for each tissue type and species to assess tissue- and species-specific patterns of CpGi methylation.

### Identification and characterization of differentially methylated regions

Differentially methylated loci (DMLs) and differentially methylated regions (DMRs) were identified using the R package DSS (Park and Wu, 2016). For each chromosome, a BSseq object was constructed from all samples of a given tissue type and species. Differential methylation testing was performed using DMLtest with smoothing enabled (smoothing = TRUE), followed by DML identification using callDML with a minimum methylation difference threshold of 20% (delta = 0.20). DMRs were identified using callDMR with the default DSS parameters (delta = 0, p.threshold = 1 × 10^−5^, dis.merge = 100, pct.sig = 0.5, minCG = 3, minlen = 50). DMRs were identified separately for each tissue and species using the DSS package in R. Note that *P. wingei* testis data comprised 4 replicates as 1 sample failed quality control.

Following DMR calling, regions were filtered to remove the top 1% of DMRs by the ratio of region length to CpG count, which likely represent spurious calls in low-complexity or repetitive regions. Sex-biased DMRs were defined as those with a difference in DNA methylation ≥25%. This threshold was selected as a conservative effect size criterion, as larger methylation differences are more likely to represent robust group-associated methylation changes than smaller differences, which may be more susceptible to background variation (Derbel et al. 2026). Shared DMRs between species were identified as overlapping DMR pairs (using genomic interval overlap) with concordant direction of methylation difference in both species, retaining only pairs where both regions exceeded the 25% differential methylation threshold in the same direction. Species-unique DMRs were defined as sex-biased DMRs in 1 species that did not overlap any sex-biased DMR in the other species.

The genomic distribution of DMRs was characterized relative to genic features and CpG islands. Promoters were defined as the region 5 kb upstream to 500 bp downstream of the transcription start site (TSS). Because this broad definition may capture distal regulatory elements in addition to proximal promoter regions, promoter-associated DMRs are interpreted as putative regulatory regions rather than exclusively canonical promoters. Introns were inferred from exon boundaries using the longest transcript per gene. For CpG island context, shores were defined as the 2 kb flanking each island, and shelves as the 2 to 4 kb flanking region. DMR overlap with each feature class was determined using subsetByOverlaps from the GenomicRanges package. To avoid double-counting DMRs across overlapping genomic features, each DMR was assigned to a single nonoverlapping category in the priority order: promoter > exon > intron > intergenic, and CpG island > shore > shelf > open sea.

To examine the genome-wide distribution of gonad-associated methylation differences, shared gonad DMRs, defined as regions showing consistent differential methylation between ovary and testis tissues in both *P. reticulata* and *P. wingei*, were analyzed in 100 kb nonoverlapping windows across all 23 chromosomes. For each window, 2 metrics were computed: first, the fraction of the window covered by hypermethylated minus hypomethylated DMRs. We also estimated the count of hypermethylated minus hypomethylated DMRs. Genome-wide 95% confidence intervals were calculated separately for positive and negative values of each metric across all autosomal windows. Windows exceeding these thresholds in the fraction metric were considered outliers. A binomial test was additionally applied per window to assess whether the ratio of hyper- to hypomethylated DMRs deviated significantly from the genome-wide expectation, with *P*-values corrected using the Benjamini–Hochberg (BH) method. Windows flagged as outliers in both the fraction metric and the binomial test were considered high-confidence outlier regions. Gene Ontology (GO) enrichment analysis of genes overlapping outlier windows was performed using goseq with gene length bias correction.

De novo motif enrichment in DMRs was assessed using HOMER (Heinz et al. 2010), comparing hypomethylated against hypermethylated DMRs across the genome. Sequences were extracted from the *P. reticulata* reference genome using DMR coordinates, with repeat-masked sequence excluded and CpG dinucleotide content normalization applied.

### RNA-seq data processing and alignment

Samples were collected from laboratory strains of *P. reticulata* and *P. wingei* maintained under standard conditions as described above. Individuals were euthanized using a lethal dose of MS-222 and dissected to isolate gonadal tissue. For each species and sex, gonads from 5 individuals were pooled to generate a single biological replicate. A total of 3 replicate pools were generated per sex (15 individuals total for each gonad type). The individuals used for RNA-seq were independent from those used for WGBS, and methylation–expression comparisons therefore represent associations between independently sampled biological replicates rather than matched individuals. Tissues were immediately flash frozen in liquid nitrogen and stored at −80 °C until RNA extraction. Total RNA was isolated using TRIzol reagent, followed by DNase treatment using the Qiagen on-column DNase digestion protocol according to the manufacturer's instructions. DNase-treated RNA samples were submitted to Génome Québec for library preparation and sequencing.

Raw sequencing reads were first assessed for quality using FastQC (Andrews 2010). Adapters and low-quality bases were trimmed using Trimmomatic (Bolger et al 2014) in paired-end mode with a sliding-window quality cutoff of 15 and minimum read length of 50 bp. Trimmed reads were aligned to the *P. reticulata* reference genome (GCF_000633615.1) using the STAR aligner (Dobin et al 2013) in a 2-pass mode to improve detection of splice junctions. In the first pass, junctions were extracted from all samples, filtered to retain those supported by ≥40 reads, and used to regenerate the genome index for the second pass. STAR was run allowing only uniquely mapping reads (–outFilterMultimapNmax 1), and alignments were output as coordinate-sorted BAM files. Sequencing and alignment quality metrics, including read counts and mapping efficiencies for all RNA-seq libraries, are provided in Supplementary Table 3. BAM files were indexed using samtools, and gene-level counts were quantified with featureCounts (Liao et al 2014) using exon features and gene identifiers from the GTF annotation file.

### Differential gene expression analysis

Gene-level counts obtained from featureCounts were imported into R and analyzed using the edgeR package (Robinson et al 2010). Genes with low expression were filtered to retain only those with ≥1 count per million (CPM) in at least half of the samples within any experimental group. Normalization factors were calculated using the TMM method to account for library size differences, and a design matrix was constructed modeling both species (*P. reticulata* vs *P. wingei*) and tissue type (ovary vs testis), including their interaction. Dispersion estimates were obtained with estimateDisp using robust empirical Bayes shrinkage, followed by quasi-likelihood F-tests to identify differentially expressed genes (DEGs) for the main effects of tissue, species, and their interaction. Genes were considered significant at a false discovery rate (FDR) <0.05 and absolute log_2_ fold change >1. Normalized expression values (CPM) were exported for downstream visualization and further analysis.

To examine the chromosomal distribution of sex-biased genes, all chromosomes were divided into nonoverlapping 100 kb windows and the proportion of testis- and ovary-biased DEGs was calculated per window, excluding windows containing fewer than 3 genes. For each chromosome, the mean and standard error of these per-window proportions were calculated and used as chromosome-level estimates of sex-biased gene density. To assess whether the sex chromosome (chromosome 12) showed atypical enrichment or depletion of sex-biased genes relative to autosomes, Fisher's exact tests were performed separately for testis- and ovary-biased gene sets, comparing gene counts on chromosome 12 against the autosomal background. To visualize the spatial distribution of sex-biased gene enrichment along chromosome 12, per-window deviations from the autosomal mean proportion were calculated and smoothed using a rolling mean of 5 consecutive windows. Reference confidence intervals were derived from the distribution of per-chromosome mean proportions across autosomes (mean ± 1.96 SD), providing a chromosome-level benchmark against which chromosome 12 could be compared.

GO enrichment analysis was performed separately for testis- and ovary-biased DEGs, as well as for species-biased and interaction gene sets, using the goseq package (Young et al 2010), which corrects for selection bias introduced by gene length differences inherent to RNA-seq data. For the sex-biased analysis specifically, the target gene set comprised genes significant for the sex effect only (not overlapping with the species or interaction contrasts) combined with genes significant for both sex and species effects but not the interaction term, thereby focusing enrichment analysis on genes with a consistent sex-biased expression pattern across both species. The population of background genes was defined as all genes retained after RNA-seq filtering that had available GO annotations, and gene lengths were estimated as the genomic span from transcription start to end site based on the *P. reticulata* reference genome annotation (GCF_000633615.1). GO terms were retained for testing if they contained a minimum of 10 annotated genes, and a probability weighting function was fitted to model the relationship between gene length and differential expression probability. Enrichment *P*-values were corrected for multiple testing using the BH method, and GO terms were considered significantly enriched at FDR <0.05.

### DNA methylation–expression coupling

Gene annotations were obtained from the NCBI GTF file. TSSs were extracted for all annotated genes, and promoter regions were defined as 5 kb upstream and 500 bp downstream of each TSS as an initial broad intersection interval; the functionally relevant proximal promoter window is defined empirically below. DMRs were intersected with promoter regions using GenomicRanges, and promoter-overlapping DMRs were assigned to genes. Gene expression was quantified as log_2_(CPM + 1). For each gene, mean male and female expression was calculated by species. Sex-biased expression was defined as Δexpression = male − female. Promoter methylation differences were defined as Δmethylation between sexes.

Spearman correlations were computed between Δmethylation and Δexpression genome-wide, among genes identified as sex-biased DEGs using edgeR, and among non-DEGs. Directional consistency with a repression model (where increased gene expression is expected to correspond to decreased DNA methylation, and vice versa) was evaluated using binomial tests. To assess scaling effects, genes were stratified into quartiles by absolute Δexpression, and correlations were computed within each quartile. Smooth 2D density plots were used to visualize genome-wide and quartile-specific methylation–expression associations.

Although promoters were initially defined as a 5 kb window upstream of the TSS for feature annotation and exploratory analyses above, this definition was intentionally broad to ensure inclusion of distal regulatory elements in the absence of empirical constraints on the functional methylation window. This enables direct evaluation of methylation–expression coupling, providing a data-driven basis to refine the functionally relevant regulatory window, while offering a more conservative and mechanistically focused approach than broad promoter annotations used for exploratory feature assignment.

To identify the genomic window that best captures this relationship, we quantified methylation–expression coupling (Spearman *ρ*) across promoter windows spanning 1 to 10 kb upstream of the TSS. The inverse relationship between promoter methylation and gene expression was strongest in proximal regions and was markedly attenuated beyond ∼2 kb upstream. Based on this empirical pattern, we defined the proximal promoter as 2 kb upstream to 500 bp downstream of the TSS for all subsequent coupling analyses. This refinement does not invalidate the broader 5 kb annotation used for exploratory feature mapping, but instead reflects a more precise resolution of the functionally relevant regulatory window for methylation–expression interactions.

Gene body methylation was examined across 4 feature classes—whole gene body, first exon, internal exons (exon ≥ 2), and introns—because these regions show functionally distinct methylation dynamics: gene body methylation broadly correlates with transcriptional elongation, first-exon methylation is linked to co-transcriptional splicing regulation, and intronic methylation is thought to suppress cryptic internal promoters. Separating these features allows the directionality and magnitude of coupling to be interpreted in its appropriate functional context.

Spearman rank correlation between Δmethylation and Δexpression was chosen as a nonparametric measure robust to outliers and the nonnormal distributions typical of methylation difference data. Correlations were computed for the full dataset and stratified by DEG status, species, and chromosomal location (chromosome 12 vs autosomes), with BH FDR correction applied across strata. To test whether the strength of methylation–expression coupling differed between *P. reticulata* and *P. wingei*, species-specific Spearman *ρ* values were compared using a Fisher *z*-transformation.

Per-pair coupling strength was defined as |Δmethylation| × |Δexpression|, capturing combined effect size across both datasets. The top 5% of pairs by coupling strength were carried forward for GO enrichment using goseq. Enrichment was tested separately for male-biased, female-biased, and chromosome 12–specific coupled genes (FDR < 0.05, BH correction; minimum 10 annotated genes per GO term).

To assess whether methylation–expression coupling shows regional enrichment, we implemented a sliding-window analysis across all 23 chromosomes. Mean coupling strength was computed in 3 Mb windows stepped at 500 kb intervals, requiring a minimum of 5 DMR–gene pairs per window. For each window, an empirical null distribution was generated by drawing, with replacement, the same number of loci as observed from the full autosomal pool of the corresponding sex-bias class (1,000 permutations per window). This autosome-matched null tests whether local coupling strength exceeds the genome-wide autosomal expectation rather than the chromosome-specific background. Empirical *P*-values were computed as the proportion of permuted means exceeding the observed window mean, and FDR correction was applied across all windows genome-wide using the BH method.

## Results

Whole-genome CpG methylation levels were broadly consistent across tissues and species, ranging from 66.3% to 76.8% mean methylation across sample groups (Fig. 1a). Testis showed markedly elevated methylation relative to all other tissues in both species, with mean whole-genome methylation of 76.8% in *P. reticulata* and 75.1% in *P. wingei*, compared to 66.3% to 68.8% across somatic and ovary tissues. Within somatic tissues, methylation levels were highly consistent across groups and species. PCA of per-CpG methylation revealed that the testis contributed the most variance in DNA methylation patterns (33.5%, PC1), suggesting a highly specialized DNA methylation pattern in the testis compared to other tissues (Fig. 1, b and c). Species-specific DNA methylation patterns were the second largest factor contributing to the variance (26.7%, PC2) in DNA methylation patterns (Fig. 1b). Differences in tissue-specific patterns between ovary and muscle tissue accounted for 6.1% (PC3) of the variance (Fig. 1c).

**Fig. 1.**
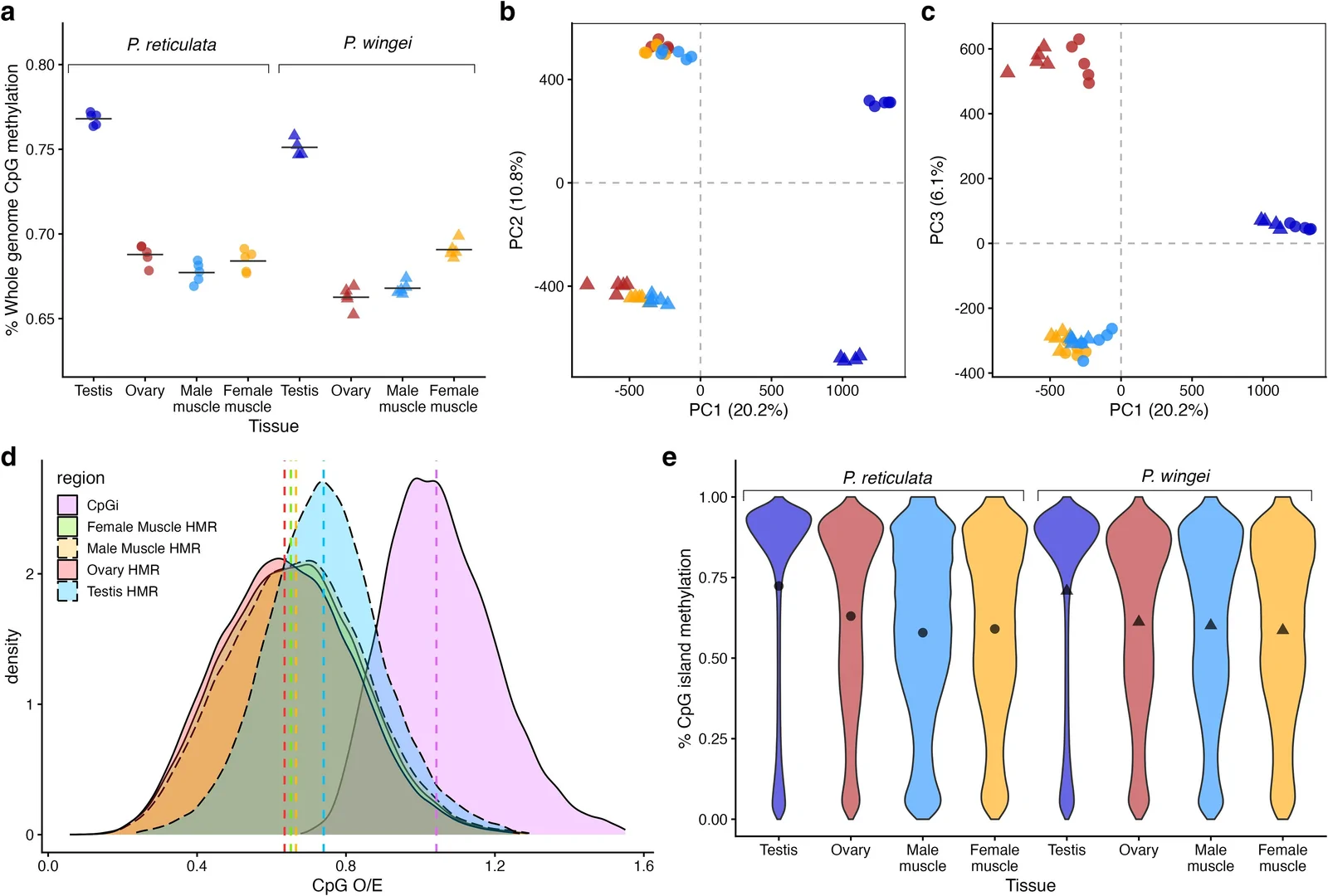
Genome-wide DNA methylation profiles across tissues and species. a) Mean whole-genome CpG methylation per individual across 4 tissue types in *P. reticulata* and *P. wingei*. Horizontal bars indicate the group mean. b, c) PCA of genome-wide CpG methylation showing PC1 vs PC2 (b) and PC1 vs PC3 (c), with percent variance explained on each axis. Points represent individual samples, colored by tissue and shaped by species. d) Density distributions of CpG O/E for annotated CpG islands and tissue-specific HMRs from each tissue type in *P. reticulata*. Dashed vertical lines indicate group medians. e) Distribution of per-island mean CpG methylation across tissues in both species, shown as violin plots with cross-sample means overlaid as points. In all panels, circles indicate *P. reticulata* and triangles indicate *P. wingei*; colors denote tissue type (testis: dark blue; ovary: red; male muscle: light blue; female muscle: orange).

Functional DMRs are frequently associated with CpG islands, particularly within promoter regions where methylation state can influence gene expression. However, commonly used CpG island identification algorithms were developed using mammalian promoter criteria and may not fully capture CpG-rich regulatory architecture in nonmammalian vertebrates (Illingworth and Bird 2009; Long et al. 2013). To address this limitation, we employed a hybrid approach. We compiled a de novo CpGi annotation for the *P. reticulata* genome by combining predictions from a HMM with empirically identified HMRs from testis, ovary, and muscle tissues, yielding a final set of 13,937 CpG islands. Consistent with canonical CpGi characteristics, these regions showed elevated CpG O/E ratios relative to tissue-specific HMRs (median CpG O/E = 1.04 for HMM CpGi vs 0.635 to 0.740 across HMR sets), confirming that the de novo annotation successfully captured CpG-rich, HMRs. Tissue-specific HMRs showed lower CpG O/E ratios, with testis HMRs (median = 0.740) being notably higher than somatic tissue HMRs (median = 0.635 to 0.666), suggesting a distinct sequence composition of testis-specific HMRs (Fig. 1d).

Having defined a consolidated CpGi annotation set, we next characterized CpGi methylation across tissues to assess how methylation state varies within putative regulatory regions. CpGi methylation was substantially lower than whole-genome methylation in all groups, as expected given that CpGi are canonically hypomethylated. Nevertheless, a clear tissue-specific pattern was evident, with testis showing the highest median CpGi methylation in both *P. reticulata* (median = 87.1%) and *P. wingei* (median = 85.2%), while somatic tissues and ovary showed considerably lower and more variable CpGi methylation (median = 60.9% to 70.6%; Fig. 1e). The broad distribution of CpGi methylation values across tissues, reflected in large IQR values (0.27 to 0.44), indicates substantial heterogeneity in island-level methylation within each tissue type, consistent with a mixture of constitutively hypomethylated and tissue-specifically methylated islands.

### Sex-biased DMRs

Differential methylation analysis identified 170,621 and 162,604 DMRs between testis and ovary tissue in *P. reticulata* and *P. wingei* respectively, of which 107,696 and 109,404 met the threshold for differential methylation (|diff.Methy| ≥ 0.25; Table 1). The majority of gonad-associated DMRs showed reduced methylation in ovary relative to testis, with 89,335 and 91,383 female-hypomethylated DMRs and 18,361 and 18,021 male-hypomethylated DMRs identified in *P. reticulata* and *P. wingei*, respectively. The vast majority of DMRs between ovary and testis were shared between species, with 80,923 overlapping regions showing concordant direction of methylation difference in both species (75.1% and 74.0% of sex-biased DMRs in *P. reticulata* and *P. wingei*, respectively), while 28,356 and 29,382 regions were species-unique.

**Table 1.** Summary of sex-biased DMRs across tissues and species.

Tissue	Species	Total DMRs	Sex-biased DMRs	Shared DMRs	Unique DMRs	Proportion shared	Proportion unique
Gonad	*P. reticulata*	170,621	107,696	80,923	28,356	0.751	0.263
Gonad	*P. wingei*	162,604	109,404	80,923	29,382	0.740	0.269
Muscle	*P. reticulata*	8,185	2,121	1	2,120	0.000	1.000
Muscle	*P. wingei*	57	57	1	56	0.018	0.982

Sex-biased DMRs in muscle tissue were far less abundant, with only a single shared DMR identified across both species and 2,121 and 57 species-unique DMRs in *P. reticulata* and *P. wingei*, respectively. These comprised 692 male-hypomethylated and 1,429 female-hypomethylated DMRs in *P. reticulata*, and 31 male-hypomethylated and 26 female-hypomethylated DMRs in *P. wingei*. To investigate the marked difference in sex-biased muscle DMR abundance between species, we compared several characteristics of the muscle methylation data. Dispersion estimates and the magnitude of sex-biased methylation differences at individual CpG sites were nearly identical (median |Δmethylation| = 0.020 in *P. reticulata* vs 0.019 in *P. wingei*), indicating that differences in DMR abundance were unlikely to reflect differences in variance or single-CpG effect sizes. *P. wingei* muscle contained more individually significant DML prior to region calling (1,476 vs 488 in *P. reticulata*). However, the DMRs identified in *P. wingei* muscle were substantially longer (median 322 vs 147 bp) and contained more CpGs (median 30 vs 19) than those identified in *P. reticulata*, indicating that sex-biased methylation in *P. wingei* is concentrated in fewer, larger DMRs. Together, these results suggest that the marked difference in DMR abundance reflects differences in the genomic organization of sex-biased methylation between species, with *P. wingei* exhibiting fewer, larger DMRs and *P. reticulata* exhibiting many more, smaller DMRs distributed throughout the genome.

Across both tissues and species, sex-biased DMRs were distributed predominantly in intergenic regions (∼40%) and introns (∼30% to 32%), with smaller contributions from promoters (∼15% to 17%) and exons (∼11% to 13%), and this pattern was broadly consistent across shared and species-unique DMR sets (Fig. 2a). Shared gonad DMRs were more enriched at CpG islands (38.3%) relative to species-unique gonad DMRs in either species (∼26% to 29%), and species-unique muscle DMRs showed the strongest CpG island enrichment (∼50% to 52%) across all comparisons (Fig. 2b).

**Fig. 2.**
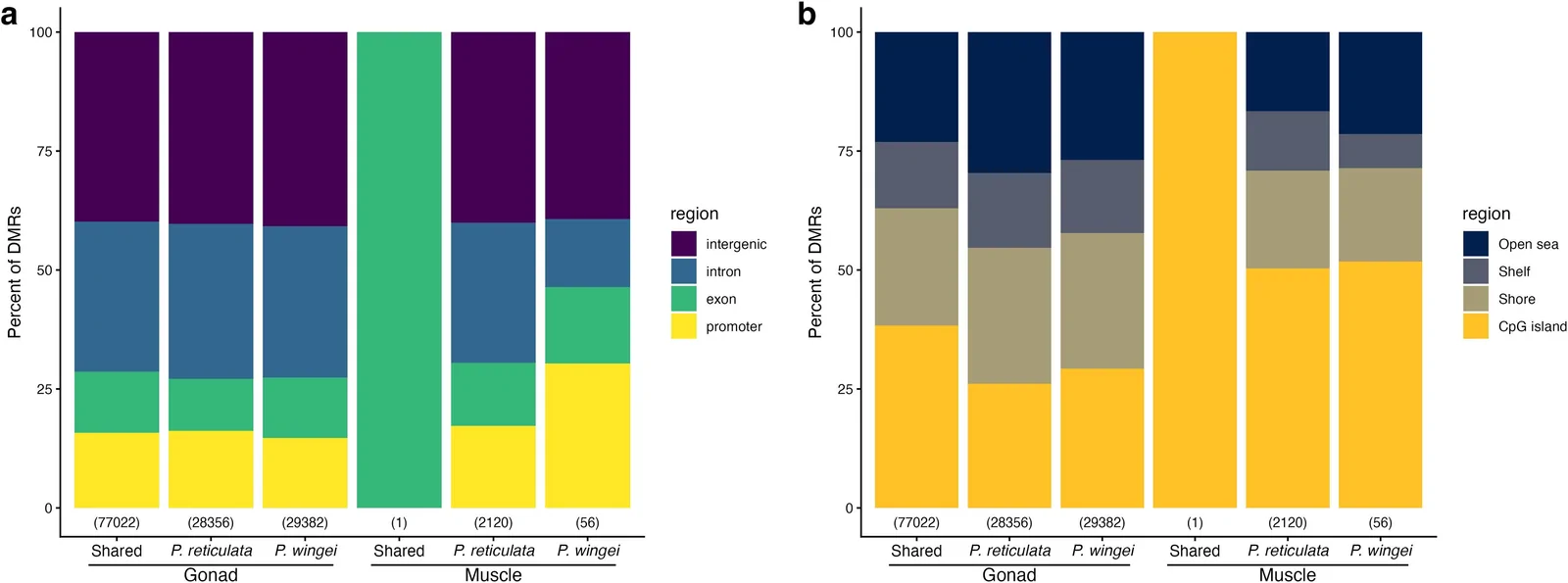
Genomic distribution of sex-biased DMRs. a) Proportion of sex-biased DMRs overlapping genic features for shared and species-specific DMR sets in gonad and muscle tissue. DMRs were assigned to a single nonoverlapping category in the priority order: promoter > exon > intron > intergenic. b) Proportion of sex-biased DMRs overlapping CpG island contexts for the same DMR sets. DMRs were assigned to CpG island, shore (±2 kb), shelf (±4 kb), or open sea in priority order. Numbers in parentheses below each bar indicate the total number of DMRs in that set. Shared DMRs are those with concordant direction of sex bias in both species; species-specific DMRs are those not overlapping any DMR in the other species.

To examine the genome-wide spatial distribution of sex-biased methylation, shared gonad DMRs were analyzed in 100 kb windows across all 23 linkage groups using 2 complementary approaches: a fraction-difference metric with genome-wide 95% CI outlier thresholds, and a binomial test of DMR directionality per window (Fig. 3a). Windows flagged by both metrics simultaneously were considered the most robust signals of localized sex-biased methylation. Here, “hypomethylated-dominant” windows refer to regions in which the majority of DMRs showed reduced methylation in one sex relative to the other, with the direction (female- or male-hypomethylated) indicating the sex with lower methylation levels. Across 6,955 windows genome-wide, 279 exceeded the upper CI threshold (female-hypomethylated-dominant) and 68 exceeded the lower threshold (male-hypomethylated-dominant). Notably, significant binomial windows occurred almost exclusively in the male-hypomethylated direction, with the single exception of 1 window on chromosome 16. Forty-eight outlier windows were identified by both metrics simultaneously. Chromosomes 16, 7, and 13 had the highest number of extreme fraction windows (26, 24, and 22, respectively).

**Fig. 3.**
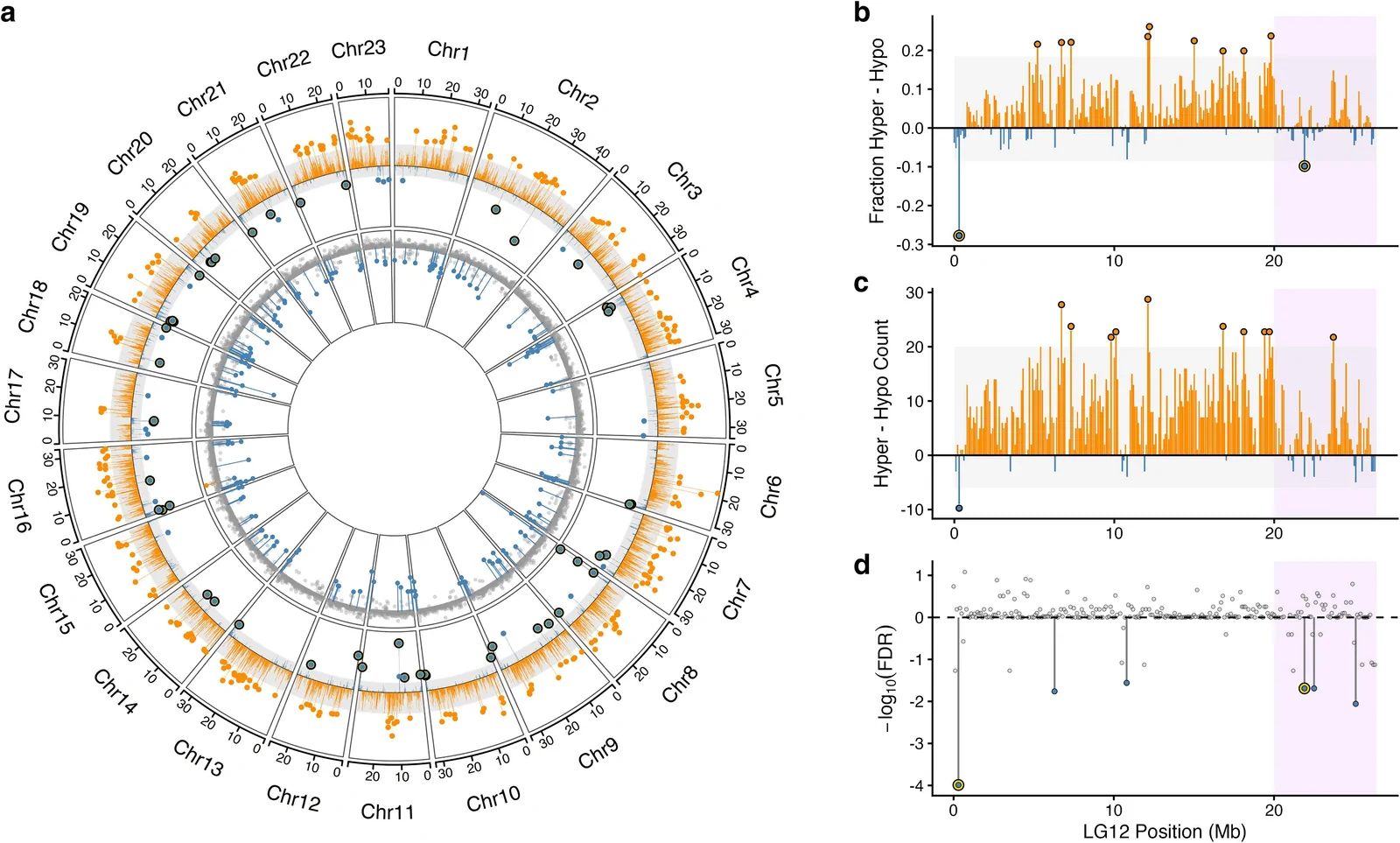
Genome-wide distribution of DMRs across linkage groups. a) Circos plot depicting DMR density and directionality across all 23 chromosomes. The outer track shows the difference in fraction of hypermethylated minus hypomethylated windows per 5 Mb genomic bin, with orange bars indicating a female hypomethylation bias and blue bars indicate a male hypomethylation bias. The gray shaded band represents the 95% confidence interval derived from the genome-wide distribution of absolute differences. Windows exceeding the confidence interval are marked with filled circles (orange or blue), and windows that additionally exhibit a statistically significant binomial enrichment are highlighted with gold-filled circles. The inner track displays the signed −log_10_(FDR) from binomial tests of DMR directionality; positive values (orange) indicate significant hypermethylation enrichment and negative values (blue) indicate significant hypomethylation enrichment, with nonsignificant windows shown in gray. (b to d) Detailed view of chromosome 12. b) Per-window fraction of hypermethylated minus hypomethylated DMRs plotted across chromosome 12 (Mb). Bar colors follow the same orange/blue convention as in (a); small filled circles denote windows exceeding the genome-wide 95% confidence interval, and gold circles highlight windows that are both extreme and statistically significant. c) Per-window count difference (hypermethylated minus hypomethylated DMRs) across chromosome 12, with filled circles marking windows beyond the 95% confidence interval. d) Signed −log_10_(FDR) from binomial tests across chromosome 12. Lollipops denote significant windows (FDR < 0.05), colored by direction of enrichment (orange: hypermethylation; blue: hypomethylation); nonsignificant windows are shown in gray. Gold circles highlight windows satisfying both the extreme fraction criterion and binomial significance. The region shaded in purple in panels (b to d) denotes the region of chromosome 12 with suppressed recombination. Genomic position is given in megabases (Mb).

On the sex chromosome, chromosome12, 11 of 264 windows exceeded the genome-wide fraction CI threshold (9 female-hypomethylated-dominant, 2 male-hypomethylated-dominant), and 6 windows were significant in the binomial test, all in the male-hypomethylated direction. Two outlier windows were identified by both metrics simultaneously, located near the proximal (0.3 Mb) and distal (21.9 Mb) ends of the chromosome (Fig. 3, b to d). Because all analyses were performed using a female-derived *P. reticulata* reference assembly, we evaluated whether reference-associated mapping bias could influence interpretation of sex-linked methylation patterns. Comparison of male coverage across chromosome 12 revealed similar patterns between species across most of the chromosome, with divergence restricted primarily to the inferred sex-determining region (SDR) at the distal end of chromosome 12 (Supplementary Fig. 2). This localized shift is consistent with expected differences in X- and Y-linked sequence composition within the SDR rather than a genome-wide species-specific alignment bias.

Importantly, chromosome 12 did not exhibit elevated DMR density relative to autosomes (Supplementary Fig. 1), indicating that reference-associated effects do not broadly explain the observed distribution of sex-biased methylation. Z-scores for chromosome 12 were within 1 standard deviation of the autosomal mean for both hypermethylated (mean count = 9.38, *z* = −0.676; mean fraction = 0.0706, *z* = −0.756) and hypomethylated (mean count = 1.87, *z* = −0.111; mean fraction = 0.0220, *z* = −0.368) DMRs. Although Grubbs’ tests identified a significant outlier for hypomethylated DMR density (count: *P* = 0.0129; fraction: *P* = 0.0438), this signal was attributable to chromosome 19, which ranked first among all chromosomes by both mean DMR count and mean fraction coverage. For hypermethylated DMRs, Grubbs’ tests detected no significant outlier (count: *P* = 0.118; fraction: *P* = 0.323). Chromosome 12 did not approach outlier status in either direction, indicating that it does not exhibit unusual enrichment for DMR density relative to autosomes.

To characterize the biological processes associated with regions of extreme sex-biased methylation, GO enrichment analysis was performed on genes overlapping fraction CI outlier windows across the full genome. Genes in male-hypomethylated outlier windows were strongly enriched for key developmental processes, including embryonic skeletal system morphogenesis (FDR = 5.4 × 10^−12^), DNA-binding transcription factor activity (FDR = 6.1 × 10^−10^), regulation of DNA-templated transcription (FDR = 6.1 × 10^−10^), and anterior/posterior pattern specification (FDR = 8.9 × 10^−10^). Genes in female-hypomethylated outlier windows were enriched for a more diverse set of terms including positive regulation of multicellular organismal process (FDR = 1.4 × 10^−2^), negative regulation of canonical Wnt signaling (FDR = 3.0 × 10^−2^), and miRNA-mediated post-transcriptional gene silencing (FDR = 4.0 × 10^−2^; Supplementary Fig. 3).

Similarly, genes located within regions of chromosome 12 enriched for sex-biased DMRs, all of which were male hypomethylated, were predominantly associated with developmental processes, including several with roles in gonad development, reproductive function, or steroidogenic tissues. Notably, the gene set includes *nr5a1b*, a nuclear receptor involved in gonad differentiation and maintenance (Agrimson et al. 2022), as well as genes associated with reproductive processes such as *psmb7*, which has been linked to spermatogenesis. Several additional loci encode transcription factors or regulators of developmental pathways (eg *lmx1b-like*, *foxd1*, and *btf3*), while others participate in cellular regulatory systems including protein turnover and transcriptional machinery (eg *psmb7* and *gtf3c5*). Although the limited number of genes within these regions precludes strong functional enrichment signals, the presence of multiple transcriptional regulators together with genes implicated in gonadal development and reproductive biology suggests that sex-biased methylation on the guppy sex chromosome may be concentrated near loci involved in developmental and reproductive regulatory processes.

To further explore whether this developmental and reproductive signal extended to canonical vertebrate sex-determination genes, we examined methylation patterns at 5 loci with well-established roles in gonadal differentiation across vertebrates: *dmrt1*, *cyp19a1*, *foxl2l*, *amh*, and *sox9a* (Supplementary Fig. 4). Sex-biased DMRs overlapped the gene body or flanking region of all 5 loci in both *P. reticulata* and *P. wingei*, with the majority of these regions occurring at concordant genomic positions and in the same direction of methylation bias in both species. This signal was most extensive at *dmrt1* and *cyp19a1*, where DMRs spanned much of the gene body itself, whereas at *foxl2l* sex-biased methylation was restricted to flanking regions upstream and downstream of the gene, with no DMRs detected within the gene body. This pattern is consistent with the Conserved Epigenetic Regulation of Sex (CERS) model (Piferrer et al. 2019), which predicts conserved epigenetic regulation of key genes involved in vertebrate sexual development. To explore potential regulatory mechanisms underlying sex-biased DMRs, we performed de novo transcription factor binding site (TFBS) motif enrichment analysis using HOMER, comparing hypomethylated against hypermethylated DMRs across the genome. Motif annotations reflect HOMER database annotations and may represent transcription factor families or related motif classes. The most significantly enriched motifs in hypomethylated DMRs included motifs corresponding to GATA3 (*P* = 1 × 10^−882^, 27.97% of targets vs 18.01% background), COUP-TFII/NR2F1 (*P* = 1 × 10^−696^, 32.48% vs 22.98%), MEIS2 (*P* = 1 × 10^−514^, 73.29% vs 64.57%), TEAD2 (*P* = 1 × 10^−486^, 35.48% vs 27.19%), NR5A2 (*P* = 1 × 10^−238^, 0.57% vs 0.06%), and POU5F1 (*P* = 1 × 10^−220^, 3.22% vs 1.51%). Additional enriched motifs included Forkhead family (FOXA2), ATF4, and PAX8 binding sequences (Supplementary Fig. 5). Together, these results indicate that hypomethylated DMRs are disproportionately enriched for motifs associated with transcription factors associated with gonadal differentiation, developmental patterning, and germ cell biology.

### Sex-biased gene expression

PCA on log-transformed CPM values for all expressed genes was performed to examine overall patterns of gene expression variation among samples. The first 2 principal components captured the majority of variance in the gene expression dataset, with PC1 explaining 66.7% and PC2 explaining 13.9% of total variation. PC1 primarily separated samples by tissue type, with ovaries and testes forming distinct clusters, while PC2 largely reflected species differences between *P. reticulata* and *P. wingei*, indicating that testis has a highly specific expression profile relative to ovary while species-specific differences comprise relatively less of the variation in the data (Fig. 4a). Consistent with this observation, differential expression analysis identified 13,191 sex-biased, 4,015 species-biased, and 3,829 interaction-biased genes (FDR < 0.05, |log_2_FC| > 1). For downstream analyses, we combined genes uniquely differentially expressed between sexes (10,337) with those overlapping the species contrast but not the interaction term (1,300), yielding 11,637 genes with a consistent direction of sex-biased expression across both species (Fig. 4b). Of these, 5,300 were testis-biased and 6,337 were ovary-biased, indicating a modest excess of female-biased expression in the ovary tissue of both species.

**Fig. 4.**
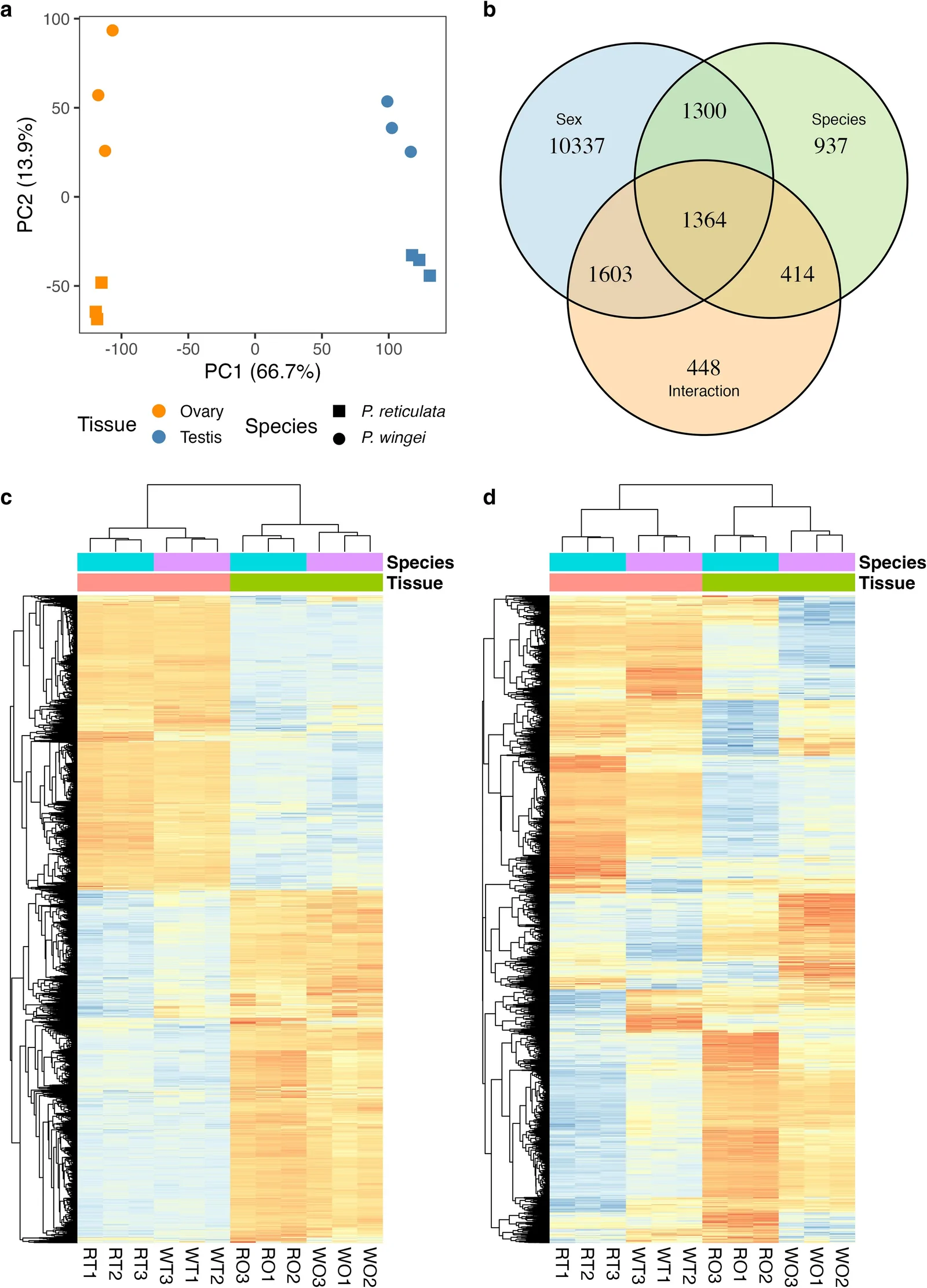
Differential gene expression between male and female gonads for *P. reticulata* and *P. wingei*. a) PCA of log-transformed CPM values across all 12 samples following TMM normalization and filtering. Each point represents 1 sample, with color indicating tissue type (orange: ovary; blue: testis) and shape indicating species (square: *P. reticulata*; circle: *P. wingei*). b) Venn diagram showing the overlap among DEGs identified for each of 3 contrasts: sex (testis vs ovary), species (*P. wingei* vs *P. reticulata*), and their interaction. DEGs were defined as FDR < 0.05 and |log_2_FC| > 1. c) Heatmap of scaled log-CPM expression for genes significant for the sex effect, excluding those also significant for the interaction term. Genes unique to the sex contrast and those shared with the species contrast (but not the interaction) are included. d) Heatmap of scaled log-CPM expression for genes significant for the species-by-sex interaction term. In both heatmaps, rows represent individual genes (unlabeled), columns represent samples clustered hierarchically, and column annotations indicate tissue type and species. Sample labels follow the convention: R = *P. reticulata*, W = *P. wingei*, O = ovary, T = testis, with the trailing number denoting the biological replicate (eg, RO1–RO3: *P. reticulata* ovary replicates 1 to 3; WT1–WT3: *P. wingei* testis replicates 1 to 3). Rows are clustered hierarchically.

GO enrichment analysis revealed functionally distinct signatures in each gene set. Testis-biased genes were strongly enriched for terms related to cilium biology and sperm motility, including cilium assembly, axoneme, and microtubule-based movement, consistent with the central role of flagellar assembly and dynein-driven motility in spermatogenesis. Ovary-biased genes were enriched for terms associated with transcriptional regulation, including RNA polymerase II cis-regulatory region binding and nucleolar components (Supplementary Table 4).

To examine the chromosomal distribution of sex-biased genes, we calculated the proportion of testis- and ovary-biased DEGs per chromosome relative to total gene content. Chromosome 12 harbors a markedly reduced proportion of testis-biased genes and falls at the extreme lower end of the chromosomal distribution (empirical *P* = 0.043; z = −3.05), indicating pronounced demasculinization (Supplementary Fig. 6). In contrast, the proportion of ovary-biased genes on chromosome 12 is elevated (*z* = 1.65) but remains within the range observed across autosomes (empirical *P* > 0.5). These patterns were not attributable to differences in chromosomal gene content, as chromosome 12 gene density (34.9 genes/Mb) falls within the autosomal range (Supplementary Fig. 5). Sliding-window analysis along chromosome 12 revealed that the proportion of testis-biased genes is reduced relative to the autosomal mean across much of the chromosome, consistent with widespread demasculinization (Supplementary Fig. 6). In contrast, ovary-biased gene density shows greater local variability and only modest elevation overall. Notably, localized regions toward the distal portion of chromosome 12 (almost equal to >20 Mb), corresponding to the putative SDR and areas of elevated X–Y divergence (Du et al. 2025; Almeida et al. 2021; Wright Alison et al. 2017), deviate from this general pattern, exhibiting relative enrichment of testis-biased genes and a corresponding reduction in ovary-biased genes.

### Distance-dependent coupling between promoter methylation and gene expression

To define the promoter window that best captures regulatory signals, we examined methylation–expression coupling as a function of distance from the TSS using 2 complementary approaches. First, DMRs were binned by their actual distance from the TSS, revealing the strongest coupling within the first kilobase (0 to 500 bp: *ρ* = −0.175; 500 bp to 1 kb: *ρ* = −0.149) and progressively weaker associations at greater distances (*ρ* = −0.050 beyond 1 kb). Second, cumulative upstream windows of increasing size showed a monotonic decline in correlation (*ρ* = −0.206 at 1 kb to −0.092 at 10 kb), consistent with dilution of the regulatory signal as more distal regions are included. These patterns were robust across species and multiple testing correction, and coupling on chromosome 12 did not differ significantly from autosomes (Wilcoxon *W* = 3,305,982, *P* = 0.163; Supplementary Fig. 7). A generalized additive model incorporating an interaction between methylation difference and distance confirmed a highly significant distance-dependent effect (*P* < 2 × 10^−16^), with model predictions showing strongest coupling proximal to the TSS, weaker associations at intermediate distances, and more variable effects distally (Supplementary Fig. 6). Together, these analyses indicate that promoter methylation exerts its strongest influence on gene expression within ∼2 kb upstream of the TSS, justifying the use of this interval as the proximal promoter region in subsequent analyses.

To determine whether sex-biased DMRs are associated with concordant differences in gene expression, we integrated the WGBS and RNA-seq datasets by linking DMRs overlapping gene promoters, defined as 2 kb upstream of the TSS and 500 bp downstream as noted above, to sex-biased expression of those genes. Across 13,810 promoter DMR–gene pairs, methylation showed a significant negative relationship with sex-biased expression (Spearman *ρ* = −0.180, FDR = 4.61 × 10^−100^; Supplementary Fig. 7), consistent with canonical promoter silencing. Coupling was stronger among sex-biased DEGs (*ρ* = −0.215, FDR = 3.51 × 10^−79^, *n* = 7,510) than non-DEGs (*ρ* = −0.109, FDR = 4.41 × 10^−18^, *n* = 6,300), and scaled monotonically with the magnitude of sex-biased expression: coupling was nonsignificant in genes with the smallest expression differences between sexes (quartile 1) and strengthened progressively through quartiles 2 to 4 (quartile 4: *ρ* = −0.243, FDR < 5.5 × 10^−47^), indicating that promoter methylation is most strongly associated with genes showing the largest sex-biased expression differences (Supplementary Fig. 7). This relationship was nearly identical between *P. reticulata* and *P. wingei* (*ρ* = −0.181 and −0.180, respectively; Fisher *z* = −0.052, *P* = 0.958), indicating that the epigenetic architecture of sex-biased expression is conserved across species.

The relationship between DNA methylation and transcription is context dependent, and associations between gene body methylation and expression can vary among gene classes and genomic features. In our analysis of sex-biased methylation, internal exon and intron DMRs showed little evidence of genome-wide coupling with sex-biased expression (*ρ* = −0.042 and −0.078, respectively), whereas first exon methylation showed a stronger negative association (*ρ* = −0.288, *n* = 7,836), comparable in magnitude to promoter methylation. Because first exons frequently overlap promoter-associated regulatory regions, this pattern is more consistent with promoter-proximal regulation than with canonical gene body methylation associated with transcriptional activity. Thus, rather than indicating that gene body methylation generally lacks regulatory associations, our results suggest that sex-biased methylation–expression coupling in guppy gonad is primarily associated with promoter-proximal regions. The consistency of this pattern between *P. reticulata* and *P. wingei* (*ρ* = −0.287 and −0.290, respectively) and its stronger association among DEGs (*ρ* = −0.323) further supports a conserved relationship between promoter-proximal methylation and sex-biased transcription.

GO enrichment analysis of the top 5% of highly coupled genes revealed consistent biological themes across both promoter and first exon DMRs. Male-biased coupled genes were enriched for meiosis and spermatogenesis-related processes, including homologous chromosome pairing at meiosis (promoter FDR = 1.6 × 10^−4^; first exon FDR = 8.8 × 10^−9^), spermatogenesis (promoter FDR = 6.6 × 10^−3^; first exon FDR = 3.9 × 10^−4^), and microtubule-based processes including axoneme and dynein complex components associated with sperm motility. Female-biased coupled genes were enriched for sperm–egg interaction terms, including acrosin binding and binding of sperm to zona pellucida (promoter FDR = 4.6 × 10^−8^; first exon FDR = 1.5 × 10^−3^), as well as egg coat formation and positive regulation of the acrosome reaction (promoter FDR = 2.5 × 10^−5^; Fig. 5a).

**Fig. 5.**
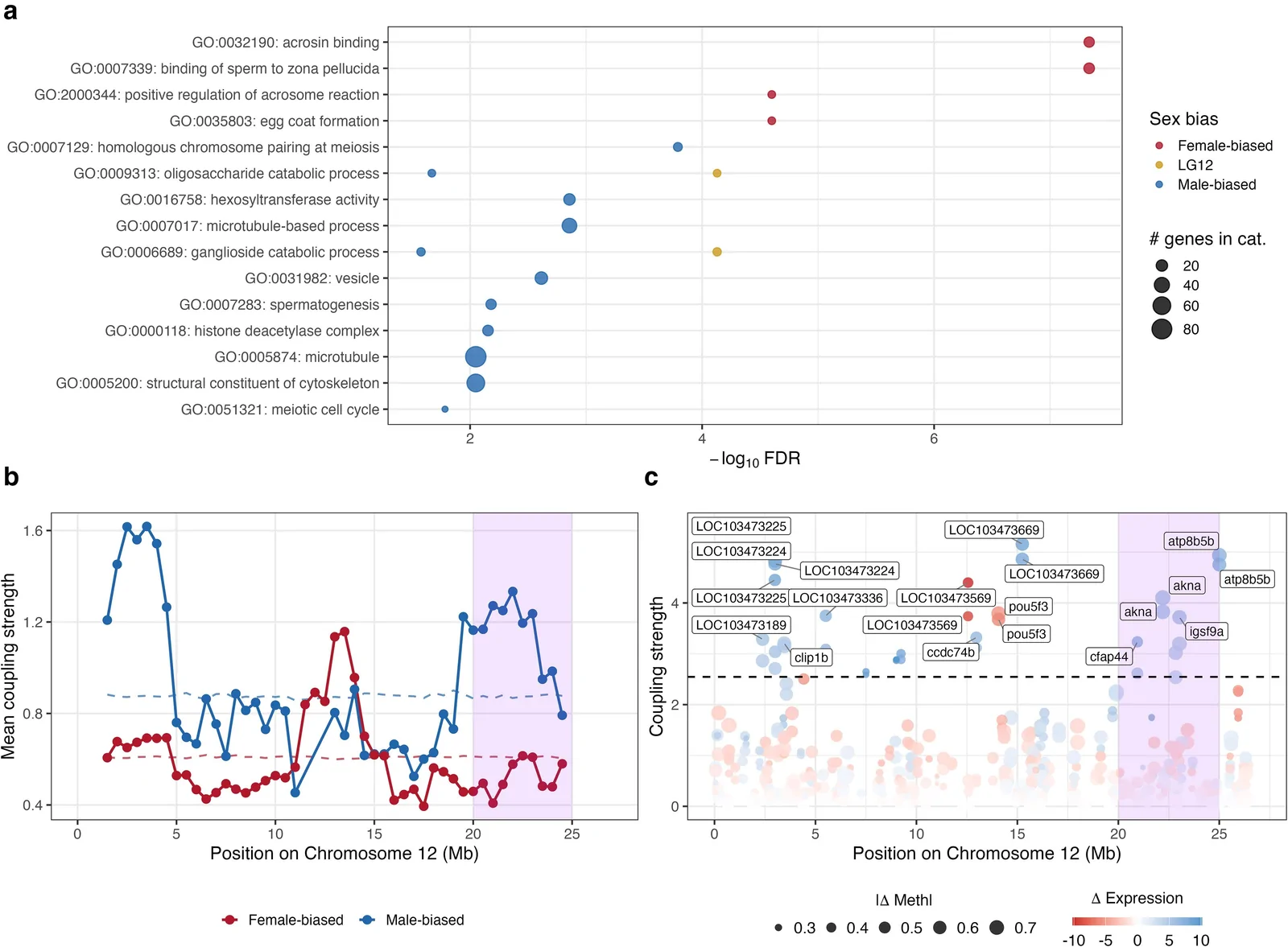
Sex-biased promoter methylation–expression coupling and functional enrichment. a) GO term enrichment for the top 5% of methylation–expression coupled genes. Dot size indicates the number of genes in each GO category, color indicates sex bias (male-biased, female-biased, or chromosome 12-specific). Only terms with FDR < 0.05 are shown. b) Sliding-window analysis of coupling strength along chromosome 12 (3 Mb windows, 500 kb steps). Solid lines show observed mean coupling per window for male-biased and female-biased genes; dashed lines indicate the autosome-matched null mean. Points denote windows with FDR < 0.05. c) Promoter coupling between Δmethylation and Δexpression for individual genes along chromosome 12. Point size represents absolute Δmethylation, color represents Δexpression (M − F), and labeled points highlight the top 20 strongest coupled genes. Horizontal dashed line indicates the 95th percentile of coupling strength.

To assess the chromosomal distribution and sex specificity of promoter methylation–expression coupling, we compared coupling strength on the sex chromosome (chromosome 12) to autosomes. Wilcoxon rank-sum testing indicated no significant difference between chromosome 12 and autosomes (*P* = 0.16; Supplementary Fig. 8). Within chromosome 12, the strongest male-biased coupling was observed near the proximal and distal regions, encompassing the putative SDR, whereas central regions approached the autosomal expectation. Female-biased coupling exhibited less pronounced positional variation (Fig. 5b). Highly coupled genes were broadly distributed along chromosome12, consistent with chromosome-wide rather than SDR-restricted epigenetic sex biasing. Among the top 5% of chromosome 12 genes with strong coupling, notable candidates included the sialidase family paralogs *neu3* and *neu4*; cilium-associated genes *cfap44*, *ccdc74b*, and *clip1b*; the phospholipid flippase *atp8b5b*; the sodium-glucose cotransporter *slc5a1*; the germ cell regulator *pou5f3*; the ubiquitin-related gene LOC103473189; and the transcription factors *tcf7l1* and *akna* (Fig. 5c).

## Discussion

Using a comparative epigenomic approach in 2 closely related Poeciliidae species, we identify conserved patterns of sex-biased DNA methylation across gonad and muscle tissue that provide insight into the organization of epigenetic regulatory architecture. Sex-biased DNA methylation in gonadal tissue shows strong tissue specificity, being highly structured in gonad but largely absent or nonconserved in muscle, indicating that epigenetic constraint on sex-biased regulation is concentrated in reproductive tissues rather than broadly distributed across somatic systems. Testis is characterized by a highly specialized methylation landscape, with discrete windows of pronounced male hypomethylation that are highly conserved between species and enriched near genes with key roles in developmental transcriptional regulation, consistent with strong evolutionary constraint at these loci. Across the genome, conserved methylation differences are negatively coupled to sex-biased gene expression, supporting a consistent regulatory relationship between methylation and transcription. On the sex chromosome, we identify localized regions of conserved male hypomethylation near genes implicated in gonadal differentiation and reproductive function. This indicates that, in this XY system with limited sex chromosome divergence, sex-biased epigenetic patterns are not disproportionately concentrated on the sex chromosome. Together, these results demonstrate that epigenetic regulatory architecture is both evolutionarily constrained and functionally organized around sex-biased developmental processes, while being shaped more strongly by tissue context than by chromosome-wide differentiation.

A key consideration when interpreting these results is that DMRs, DEGs, and methylation–expression coupling capture complementary layers of regulatory variation. Because these comparisons are based on ovary and testis tissues, conserved gonadal DMRs may reflect both sex-associated regulatory differences and differences in the cellular composition or functional specialization of gonadal tissues. DMRs identify loci of epigenetic divergence that may reflect both active regulation and regulatory potential, while DEGs represent downstream transcriptional output. Coupling analyses highlight loci where methylation and expression covary, identifying relationships likely to be functionally active in the sampled tissue. Conservation across species is consistent with these methylation patterns representing evolutionarily maintained features of the regulatory landscape. While some conserved DMRs are associated with ongoing transcriptional regulation in the adult gonad, others may instead reflect conserved regulatory architecture linked to developmental gene programs.

The most striking feature of the genome-wide methylation data is the highly distinctive methylation landscape of testis relative to all other tissues. This pattern is consistent with the well-documented specialization of the vertebrate testis methylome, in which globally elevated methylation levels are thought to reflect the silencing of transposable elements and repetitive sequences during spermatogenesis (Hammoud et al. 2009; Champroux et al. 2018). During spermiogenesis, extensive chromatin remodeling replaces histones with protamines, enabling tight DNA compaction in mature sperm. Dense DNA methylation is thought to facilitate this process, while discrete HMRs correspond to sites of nucleosome retention that remain accessible and are proposed to carry regulatory information into the embryo (Hammoud et al. 2009; Brykczynska et al. 2010; Carone et al. 2014; Jung et al. 2017).

Importantly, this general organization is not restricted to mammals. In teleost systems such as zebrafish, sperm methylation patterns are partially retained through early embryogenesis, and paternal epigenetic contributions can influence early developmental regulatory states (Jiang et al. 2013; Potok et al. 2013; Ortega-Recalde et al. 2019; Skvortsova et al. 2019). More broadly, comparative analyses in fish demonstrate that developmental regulatory genes are frequently associated with conserved methylation dynamics during early embryogenesis, suggesting that sperm epigenetic architecture can contribute to early developmental regulatory landscapes (Long et al. 2013; Tena et al. 2014). Together, these observations suggest that the combination of globally high methylation and localized hypomethylation observed in guppy testis reflects a conserved vertebrate feature of germline epigenome organization rather than a lineage-specific phenomenon.

Consistent with this interpretation, TFBS motif enrichment analysis shows that hypomethylated DMRs in testis are enriched for binding sites of transcription factors with established roles in gonadal differentiation and germ cell biology, including GATA3 and COUP-TFII/NR2F2 (Grote et al. 2006; Zhao et al. 2017; Petit et al. 2007), as well as MEIS2, a cofactor of HOX proteins required for developmental patterning (Amin et al. 2015; Delgado et al. 2021). Other enriched TFBS of note included NR5A2 and POU5F1 motifs, linked to Sertoli cell function, spermatogenesis, and germ cell maintenance (Kehler et al. 2004; Hinshelwood et al. 2005; Zheng et al. 2016; Agrimson et al. 2022), further supporting the localization of HMRs to regulatory regions important for germline development. More broadly, conserved male-hypomethylated DMRs near developmental regulators are consistent with a testis methylome organized around discrete regulatory regions, paralleling vertebrate sperm methylomes in which hypomethylated and nucleosome-retaining loci are enriched at developmental genes (Hammoud et al. 2009; Brykczynska et al. 2010; Carone et al. 2014). Integrating these patterns with GO enrichment of nearby genes indicates that conserved male hypomethylation in guppy testis is concentrated at regulatory regions associated with key developmental loci (Hammoud et al. 2009; Long et al. 2013; Tena et al. 2014; Champroux et al. 2018). While some of these loci may contribute to ongoing transcriptional regulation in the adult testis, others may instead represent regulatory features maintained because of their roles during earlier developmental stages (Hammoud et al. 2009; Jiang et al. 2013; Potok et al. 2013; Carone et al. 2014; Jung et al. 2017).

Sexually dimorphic DNA methylation has been documented across diverse vertebrate systems (Liu et al. 2010; Chatterjee et al. 2016; Metzger and Schulte 2018; Lundregan et al. 2026; Höglund et al. 2024; Shealy et al. 2025), but the biological context in which these patterns arise varies substantially. In species with environmental sex determination, sex-biased methylation is directly tied to environmental cues that influence sex determination (Navarro-Martín et al. 2011; Anastasiadi et al. 2018), whereas in systems with highly diverged sex chromosomes, enrichment of sex-biased methylation on the sex chromosome is often associated with gene content differences and dosage compensation (Liu et al. 2010; Metzger and Schulte 2018).

In *P. reticulata* and *P. wingei*, sex chromosomes are largely homomorphic with minimal Y degeneration. Despite the absence of extensive sequence divergence or gene loss, chromosome 12 shows a depletion of testis-biased genes relative to autosomes, consistent with demasculinization associated with X-linked transmission through females (Parisi et al. 2003; Vicoso and Charlesworth 2006; Reinius et al. 2012). Moreover, this demasculinization is broadly distributed across the chromosome rather than confined to the SDR, extending previous observations in the guppy despite limited Y degeneration (Sharma et al. 2014). However, the distal region encompassing the putative sex-determining locus represents a notable exception, showing relative enrichment of testis-biased genes. This same region is independently supported by enrichment of conserved male-hypomethylated DMRs and elevated methylation–expression coupling, suggesting coordinated regulation of testis-specific genes within a localized genomic domain. Because the SDR represents the region where female reference–associated effects would be most likely to occur given the absence of a Y-linked assembly, we also evaluated whether sequence divergence between sex chromosomes could contribute to the observed SDR-associated patterns. Male coverage comparisons across chromosome 12 revealed similar patterns between species across most of the chromosome, with divergence primarily restricted to the inferred SDR interval (∼20 to 25 Mb), consistent with localized differences between X- and Y-linked haplotypes rather than broad reference-associated mapping bias. Moreover, the concordance of independent genomic and epigenomic signals suggests that this region warrants further investigation as a potential hotspot of sex-biased regulatory variation. More broadly, the absence of chromosome-wide enrichment for sex-biased DNA methylation indicates that sex-biased epigenetic regulation occurs through discrete genomic regions rather than chromosome-wide remodeling.

The presence of demasculinization across LG12 in both *P. reticulata* and *P. wingei*, in the absence of detectable Fast-X evolution, contrasting with more derived Poeciliids such as *Poecilia picta* and *Poecilia parae* where Y degeneration is associated with accelerated X-linked sequence evolution (Darolti et al. 2023), suggests that shifts in the distribution of sex-biased gene expression can arise early in sex chromosome evolution and precede the emergence of Fast-X dynamics. However, the lack of gene movement from the X to the autosomes in *P. reticulata* and *P. wingei* (Lafond et al. 2026) suggests that this shift is due to regulatory changes rather than changes in gene content.

Genome-wide analyses of methylation–expression coupling further indicate that sex-biased epigenetic regulation is functionally associated with reproductive biology. Male-biased coupled genes are enriched for meiosis and spermatogenesis-related processes, including homologous chromosome pairing, sperm motility, and axonemal function. In contrast, female-biased coupled genes are enriched for sperm–egg interaction and fertilization. These patterns indicate that methylation is associated with sex-specific regulation of reproductive gene function in gonadal tissue.

In the distal region of chromosome 12, several genes showing strong methylation–expression coupling have well-characterized roles in sperm biology, including *cfap44* and *ccdc74b* (flagellar structure), *atp8b5b* (spermatogenesis), *slc5a1* (sperm energetics), and *neu3*/*neu4* (cell surface remodeling). The co-occurrence of multiple functionally related loci under consistent epigenetic regulation in this region suggests coordinated control of testis-specific gene expression. Notably, this occurs within a region of elevated X–Y divergence but is not restricted to it, reinforcing the interpretation that sex-biased epigenetic regulation reflects functional organization rather than a passive consequence of sequence divergence.

Comparisons between closely related species provide insight into the extent to which methylation patterns are conserved vs shaped by lineage-specific or context-dependent processes. Conserved methylation patterns may arise through multiple biological processes, including evolutionary constraint acting on regulatory architecture, conserved developmental programs, or similar cellular and germline epigenomic states. In gonadal tissue, differential methylation is highly conserved between species and concentrated at discrete regulatory regions enriched for developmental transcription factors and spermatogenesis genes. In contrast, sex-biased methylation in somatic tissue is not conserved across species, indicating that these patterns are more likely to reflect lineage-specific or context-dependent regulatory variation.

More broadly, conserved hypomethylated domains in testis are not solely associated with adult sex-specific gene expression, but appear to reflect a deeper, evolutionarily constrained organization of regulatory elements within the vertebrate germline epigenome. This organization is consistent with a model in which developmental regulatory genes are embedded within conserved epigenetic domains in sperm, consistent with a conserved regulatory function that may contribute to early developmental gene regulation.

## Supplementary Material

jkag228_Supplementary_Data

## Data Availability

Sequencing reads are available from the Genbank Sequence Read Archive (BioProject ID: PRJNA1460787). The processed data supporting this study have been deposited in the Borealis Canadian Dataverse Repository and are available at https://doi.org/10.5683/SP4/RZQDZW. Scripts used for data processing and analysis can be found at https://github.com/D-Metzger/guppyWGBS. Supplemental material available at G3 online.
