# Supplementary material for: Conserved sex-biased DNA methylation patterns target key developmental genes and reproductive function in two Poeciliidae species
Source: G3 (Bethesda). 2026 Aug 19;16(10):jkag228. doi: 10.1093/g3journal/jkag228 (PMC13643794; doi:10.1093/g3journal/jkag228)
Supplement: Supplementary file 1 [file jkag228_supplementary_data.pdf]

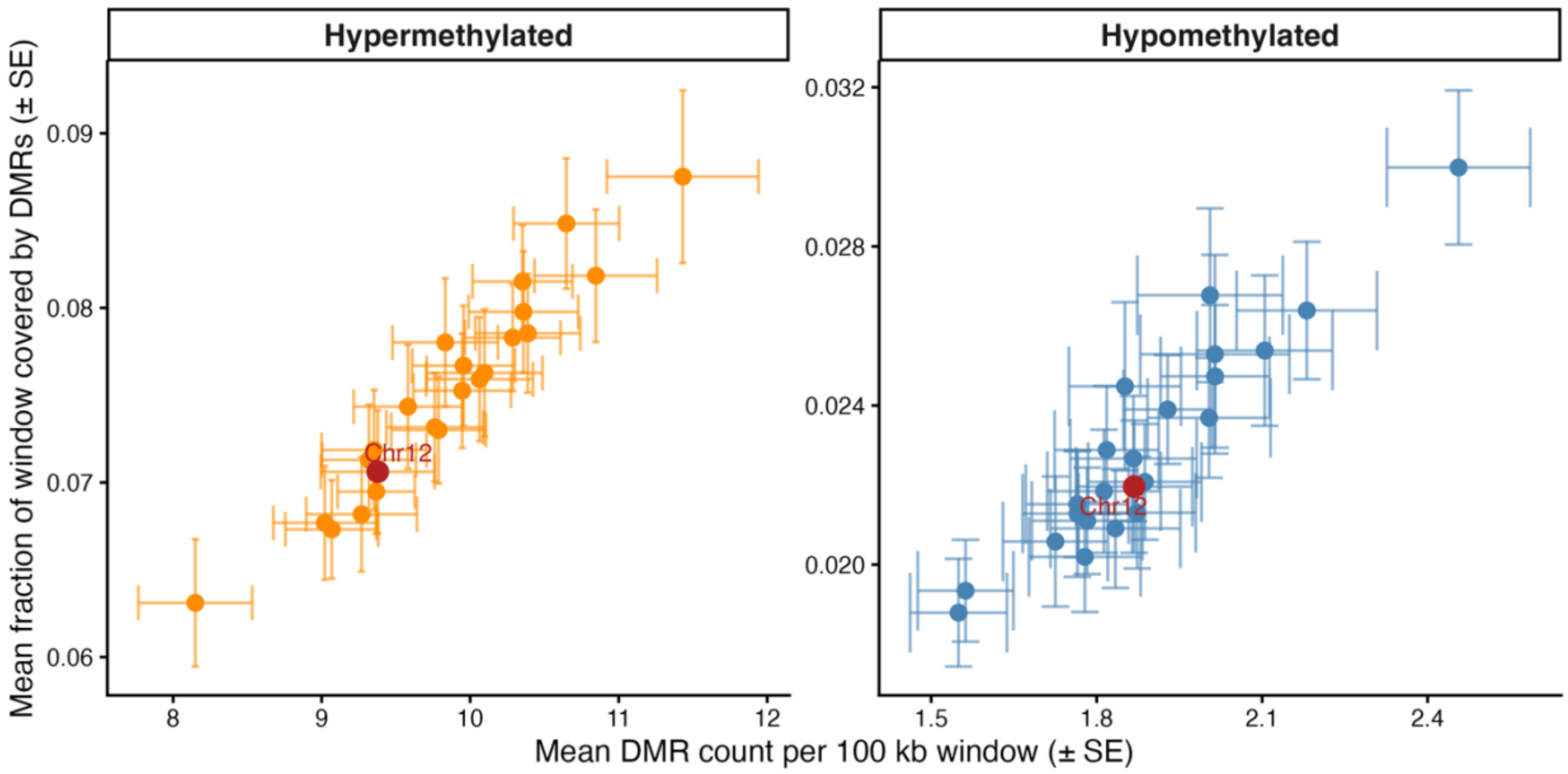

Figure S1. Chromosome-level distribution of DMR density across the genome. Scatterplots show the relationship between the mean number of differentially methylated regions (DMRs) per 100 kb window and the mean fraction of each window covered by DMRs for each chromosome. Points represent chromosome-wide averages, with horizontal and vertical error bars indicating standard error for DMR count and DMR coverage fraction, respectively. Panels are separated by methylation direction, with hypermethylated chromosomes shown in orange and hypomethylated chromosomes shown in blue. Chromosome 12 (Chr12) is highlighted in red. Chr12 did not exhibit elevated DMR density relative to the genomic background, falling within the range of autosomal variation for both hyper- and hypomethylated DMRs.

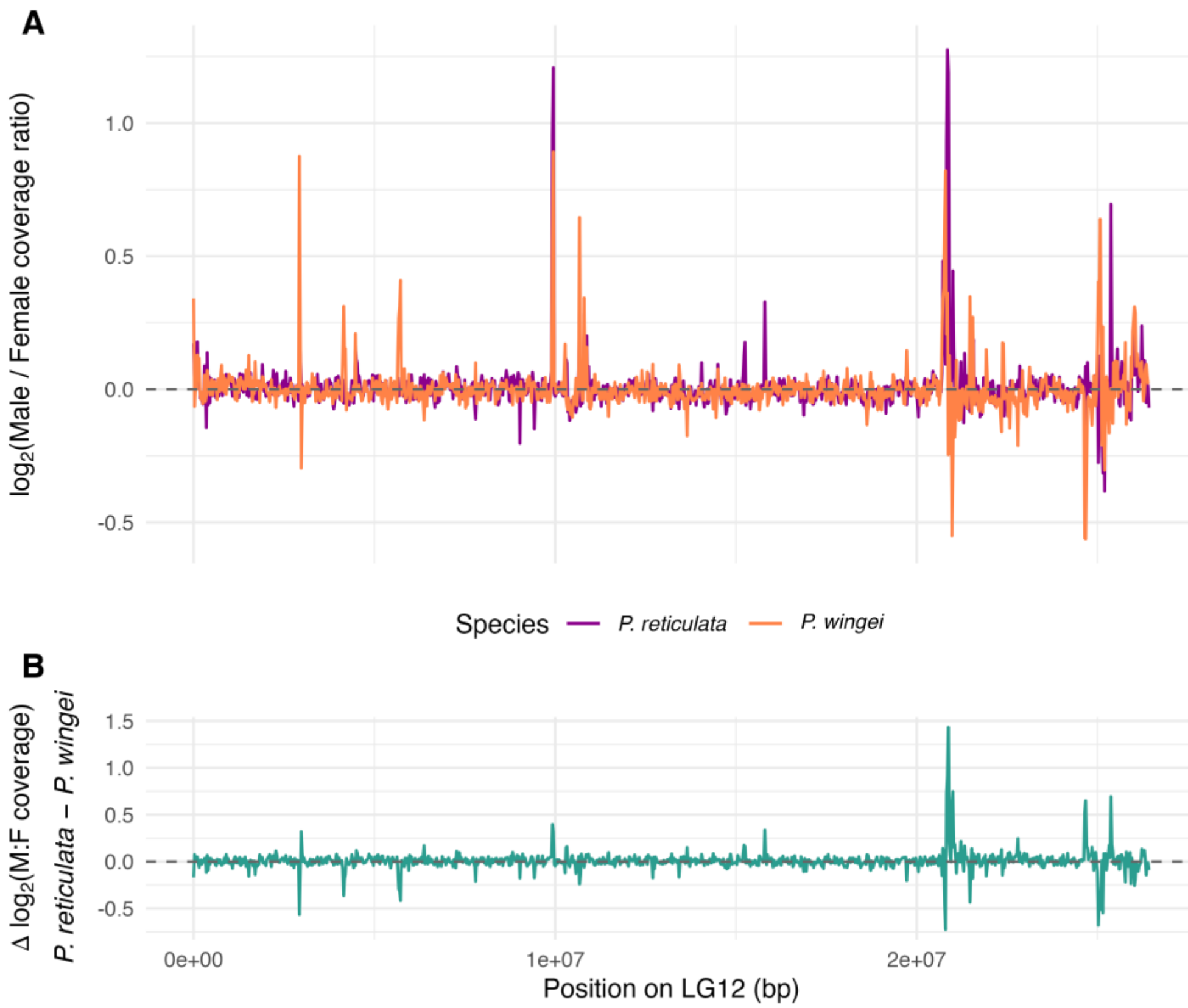

Figure S2. Male coverage ratios across Chromosome 12 in *Poecilia reticulata* and *P. wingei*. (A) Log2-transformed male:female (M:F) coverage ratios across Chromosome 12 for *P. reticulata* and *P. wingei*. Coverage ratios were calculated across 25kb genomic windows, with values near zero indicating similar male and female coverage. (B) Difference in log2(M:F) coverage ratio between species (*P. reticulata* − *P. wingei*) across Chromosome 12.

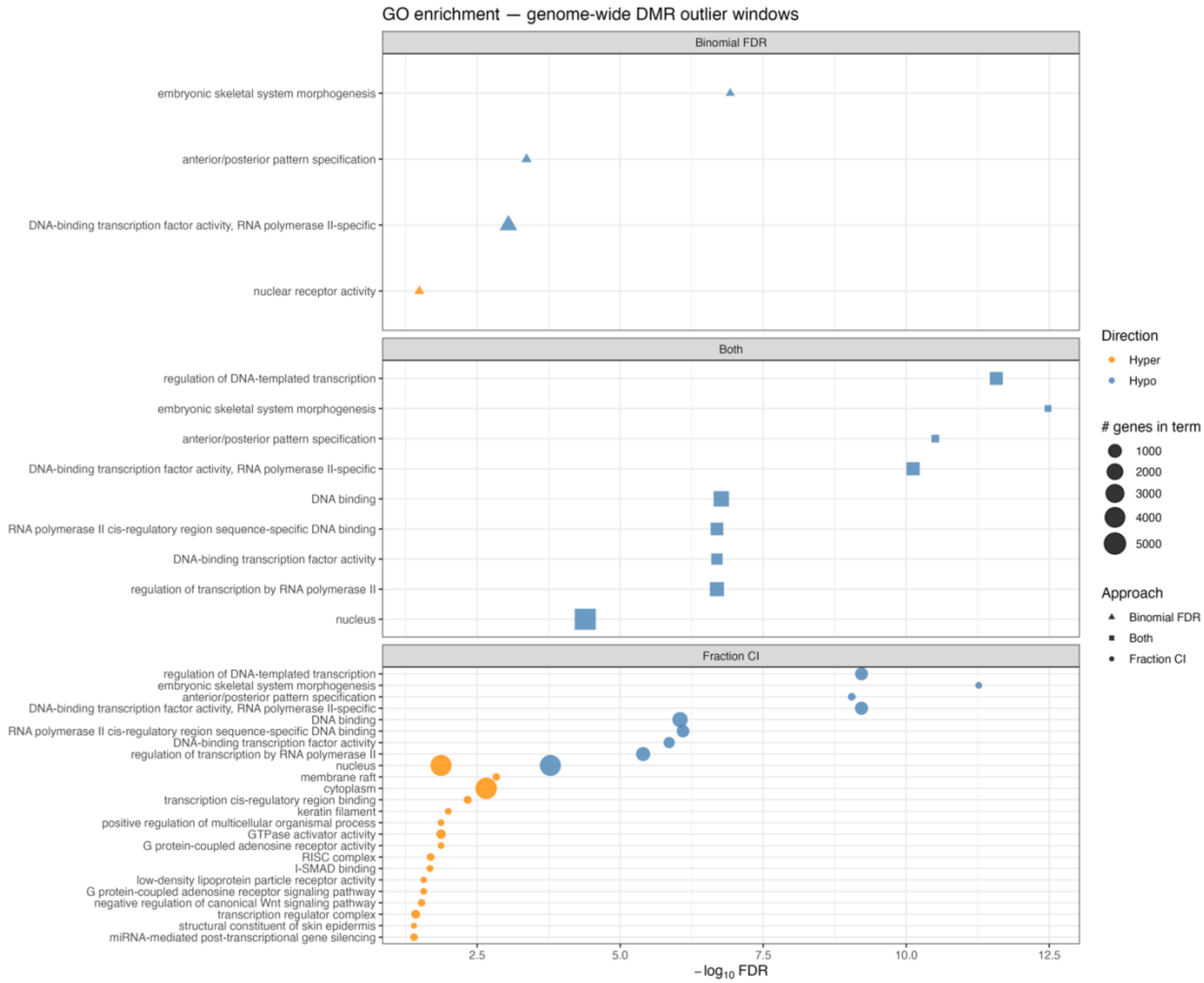

Figure S3. GO enrichment analysis of sex-biased DMRs identified by three statistical approaches: regions significant in a binomial test (upper panel, triangles), regions where the fraction of DMRs exceeded the genome-wide 95% confidence interval (lower panel, circles), and regions significant in both analyses (middle panel, squares). Orange and blue symbols indicate GO terms enriched in hypermethylated and hypomethylated male DMRs, respectively. Symbol size reflects the number of genes associated with each term.

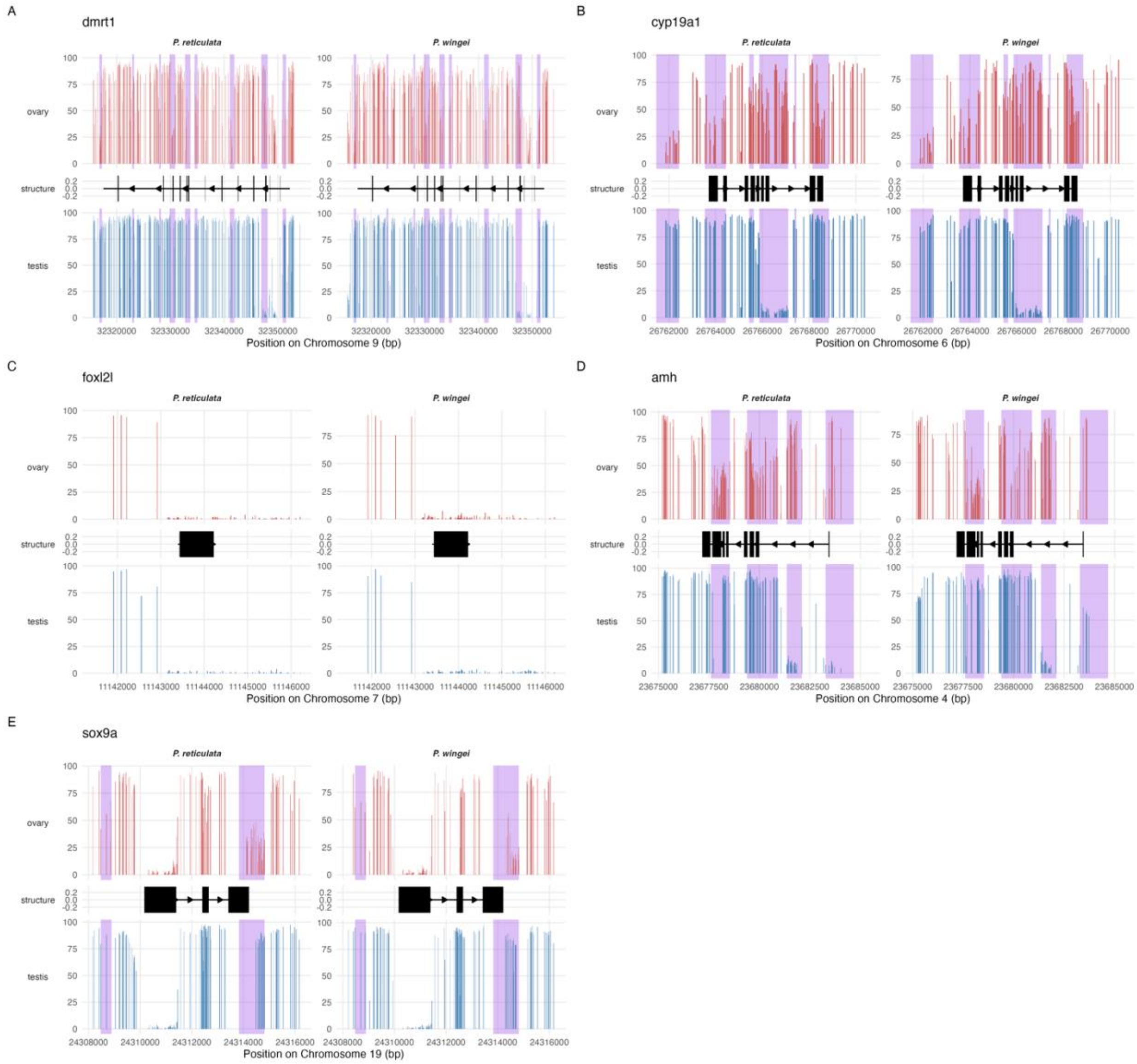

Figure S4. Sex-biased DNA methylation at canonical vertebrate sex-determination genes. Percent methylation at individual CpG sites across five genes with well-established roles in vertebrate gonadal differentiation: (A) *dmrt1* (LG9), (B) *cyp19a1* (LG6), (C) *foxl2l* (LG7), (D) *amh* (LG4), and (E) *sox9a* (LG19). For each gene, panels show mean percent methylation per CpG site in testis (blue, top) and ovary (red, bottom) for *P. reticulata* (left) and *P. wingei* (right), spanning the gene body plus 2 kb upstream and downstream. The central track depicts gene structure, with black boxes representing exons, connecting lines representing introns, and arrows indicating the direction of transcription. Shaded purple regions denote sex-biased differentially methylated regions (DMRs) shared between *P. reticulata* and *P. wingei* at concordant genomic positions and in the same direction of methylation bias

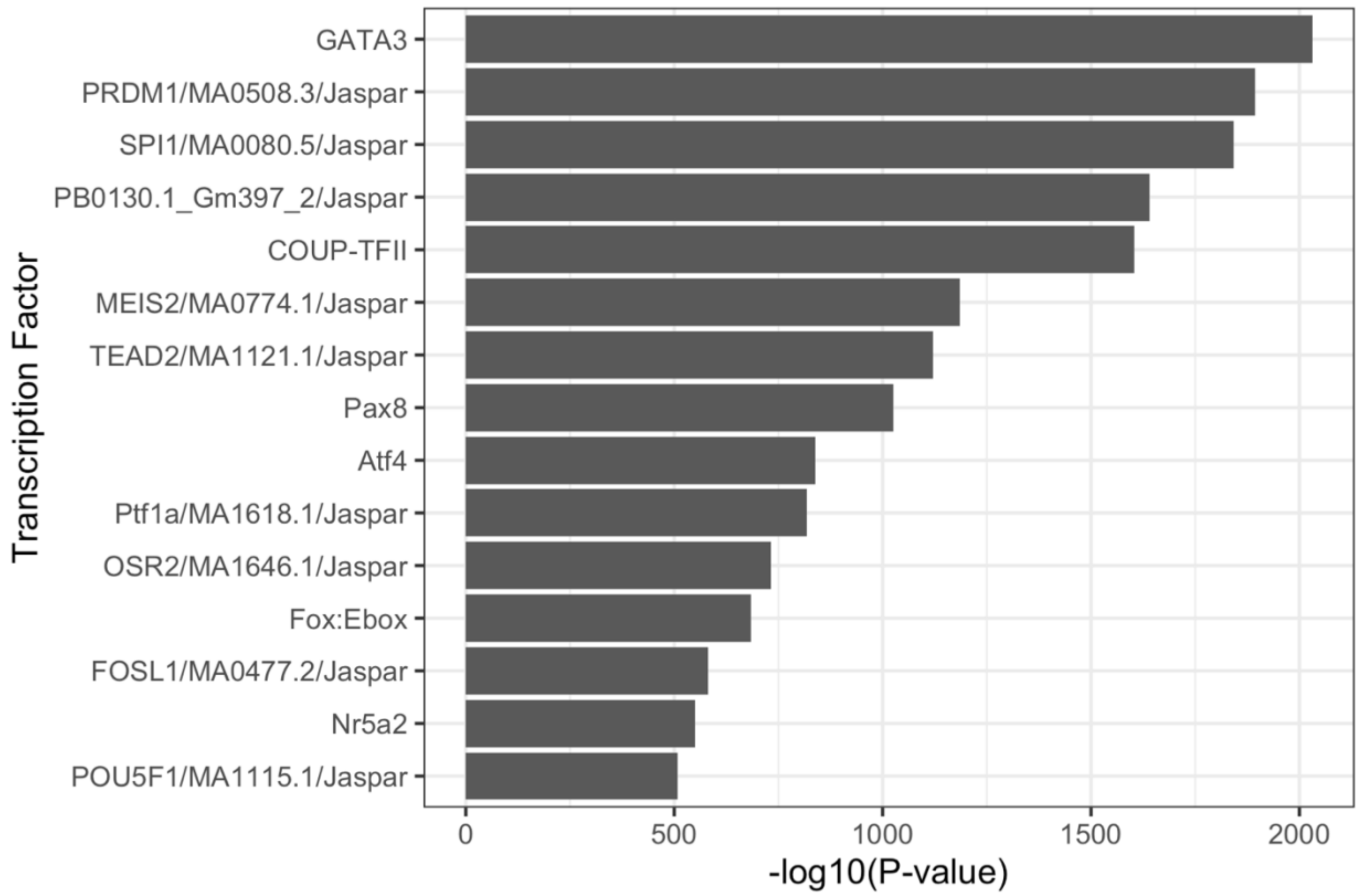

Figure S5. Transcription factor binding site enrichment from HOMER analysis.
Top enriched transcription factor binding motifs identified by HOMER are shown, ranked by −log10 p-value. Bars represent enrichment significance, and motif labels correspond to the best-matching transcription factors.

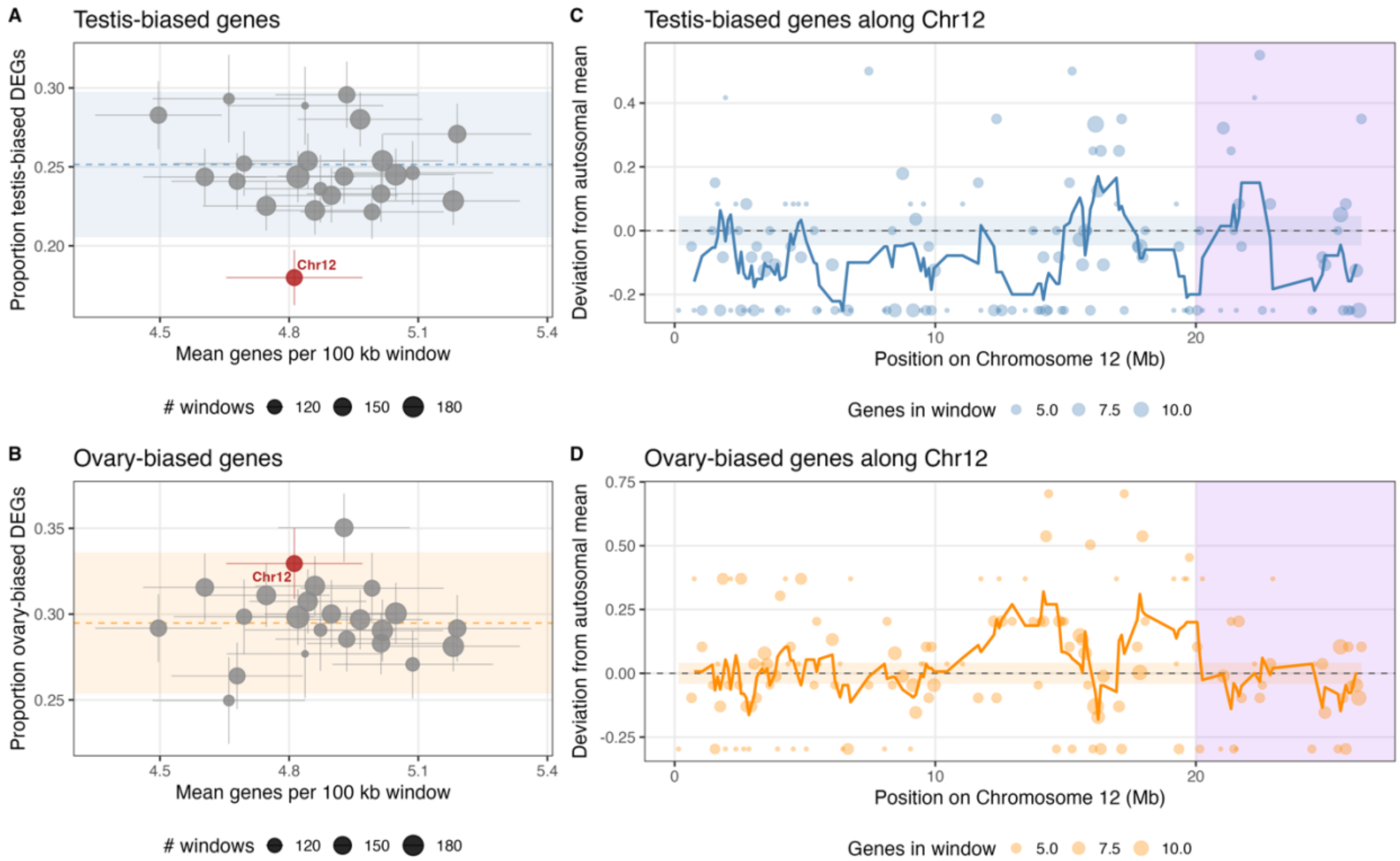

Figure S6. Chromosome 12 is depleted of testis-biased genes genome-wide but harbours localised enrichment within the sex-determining region.

(A, B) Proportion of testis-biased (A) and ovary-biased (B) DEGs per linkage group plotted against mean gene density (genes per 100 kb window), with error bars representing ± one standard error across windows. Each point represents one linkage group, with point size scaled to the number of 100 kb windows. Chromosome 12 is highlighted in red; all other linkage groups are shown in grey. The dashed horizontal line indicates the autosomal mean and the shaded band the 95% confidence interval derived from the distribution of autosomal chromosome means. **(C, D)** Deviation from the autosomal mean proportion of testis-biased (C) and ovary-biased (D) DEGs in 100 kb sliding windows along Chromosome 12, plotted against chromosomal position (Mb). Points represent individual windows sized by gene content, and the solid line shows a rolling mean across $k = 5$ windows. The blue and orange shaded bands indicate the autosomal 95% confidence interval and the dashed line marks zero deviation from the autosomal mean. The shaded purple region in panels C and D denote the area of suppressed recombination. DEGs were defined as FDR < 0.05 and $|\log_2 FC| > 1$.

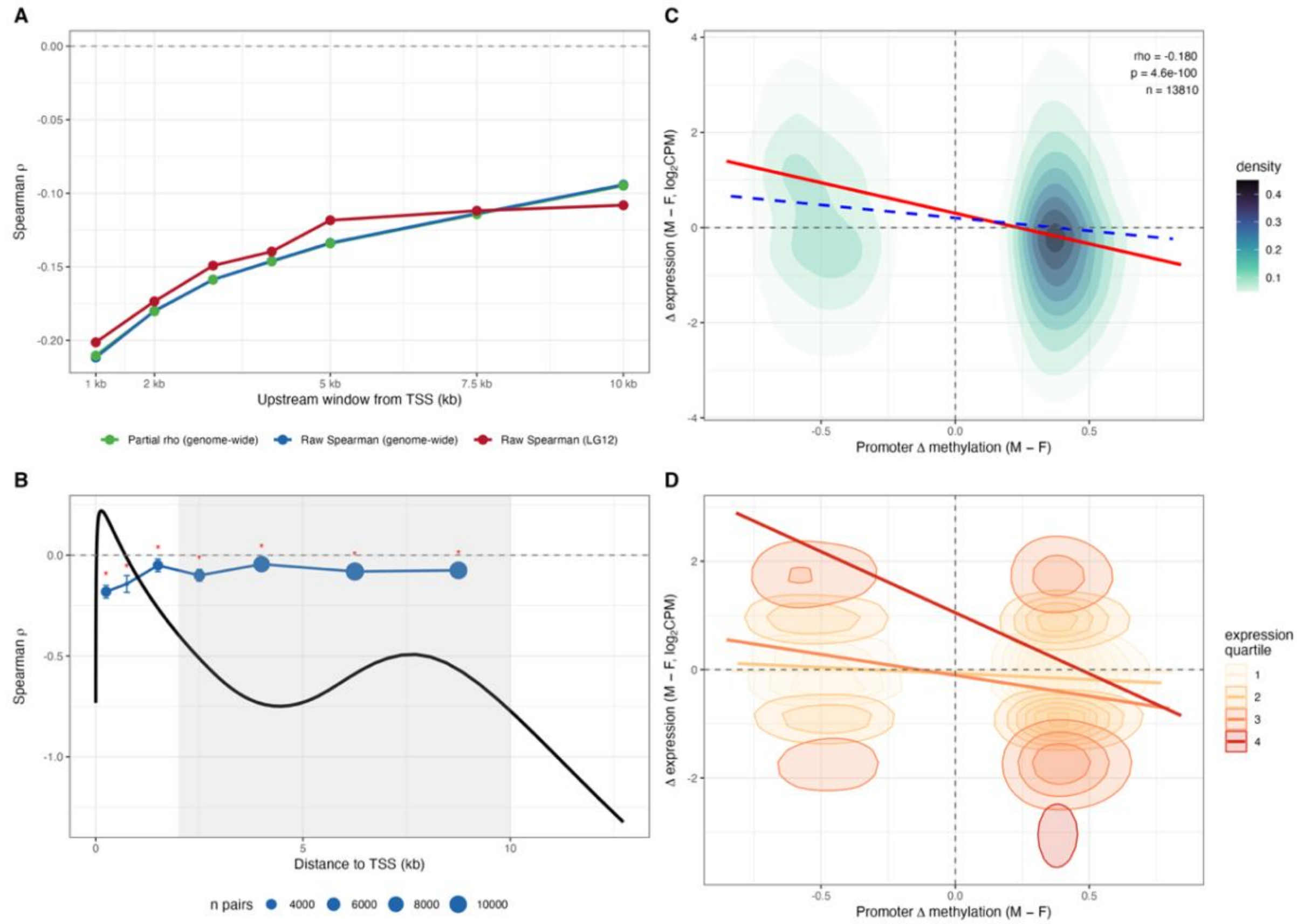

Figure S7. Spatial and effect-size relationships between promoter DNA methylation and gene expression.

(A) Spearman correlation (ρ) between promoter methylation and expression plotted across upstream windows from the transcription start site (TSS). Correlations were calculated genome-wide (raw and partial) and for linkage group 12 only. Shaded regions indicate the window considered distal (2–10 kb). (B) Distance decay of methylation–expression coupling. Points represent binned pairwise correlations with 95% confidence intervals, and point size reflects the number of gene pairs. The black line shows the continuous gradient estimated via generalized additive modeling (GAM), highlighting decreasing correlation with increasing TSS distance. Red asterisks indicate statistically significant correlations (FDR < 0.05). (C) Two-dimensional density of methylation versus expression differences (male − female) for differentially expressed genes (DEGs, red regression line) and non-DEGs (blue dashed line). Overlaid annotations report Spearman correlation, p-value, and sample size. Density shading reflects the concentration of points. (D) Density contours and linear regression of methylation–expression differences stratified by expression quartile. Each color represents a quartile of expression level, illustrating how the strength of coupling varies with baseline expression. In panels C and D, dashed lines denote zero change in methylation or expression.

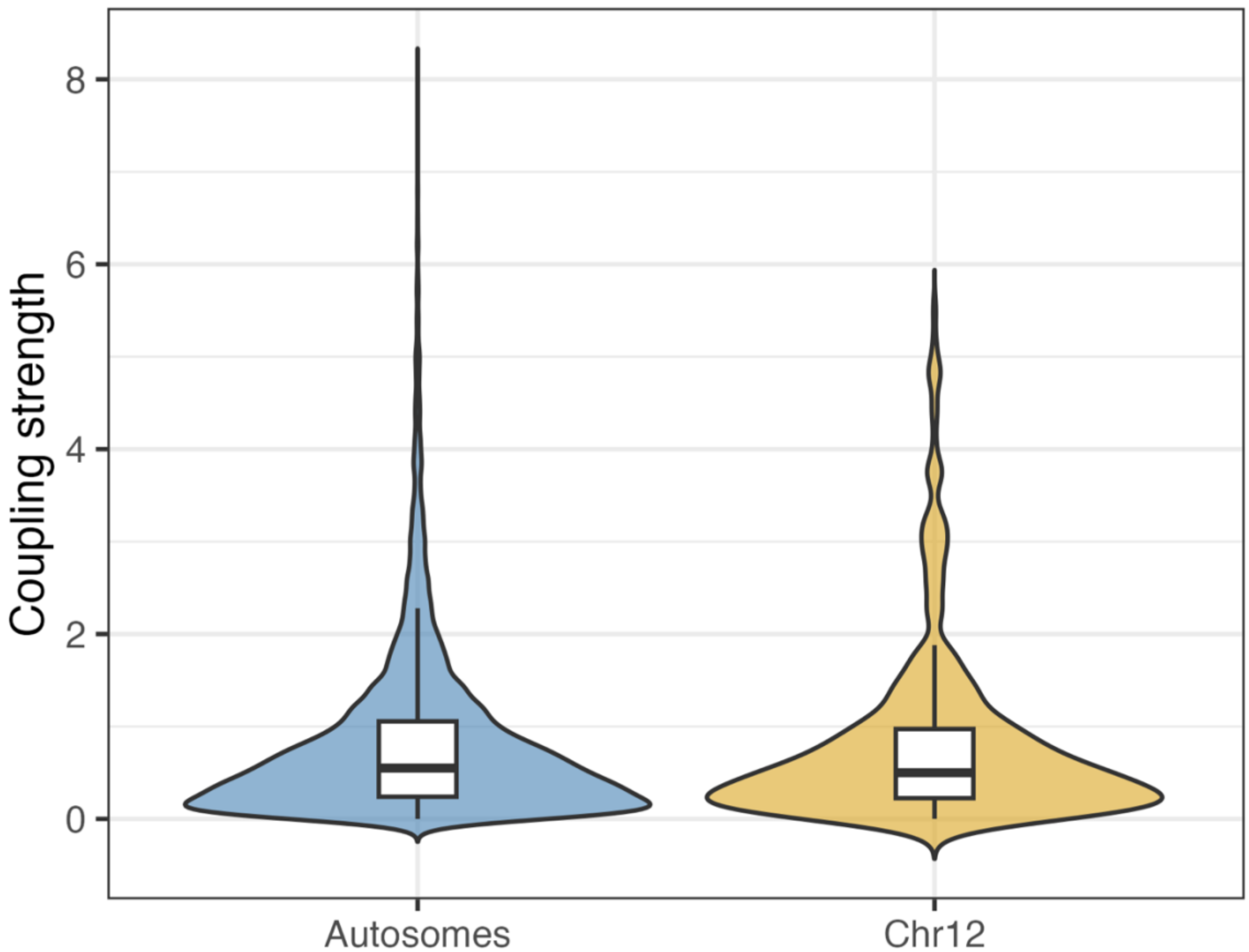

Figure S8: Violin plots display the distribution of promoter methylation–expression coupling values for genes on the sex chromosome (Chromosome 12) and autosomes; embedded boxplots indicate the median and interquartile range. A Wilcoxon rank-sum test showed no significant difference in coupling strength between Chromosome 12 and autosomal genes ($p = 0.16$).

Table S1. Sample-level WGBS sequencing and alignment quality metrics.

Sample	Species	Sex	Tissue	Total reads	% mapped	% duplicate	Median CpG depth	% CpG ≥10x
rfm1	*P. reticulata*	Female	Muscle	153030915	96.87	7.21	8	30.3
rfm2	*P. reticulata*	Female	Muscle	183676564	96.99	10.17	8	40.0
rfm3	*P. reticulata*	Female	Muscle	146556133	96.92	6.47	8	30.6
rfm4	*P. reticulata*	Female	Muscle	128500237	96.93	9.08	6	17.2
rfm5	*P. reticulata*	Female	Muscle	99966120	100.0	11.58	8	34.3
rmm1	*P. reticulata*	Male	Muscle	131488650	96.71	7.31	6	14.1
rmm2	*P. reticulata*	Male	Muscle	145056917	96.89	6.11	8	30.6
rmm3	*P. reticulata*	Male	Muscle	160901120	96.92	6.72	9	41.4
rmm4	*P. reticulata*	Male	Muscle	127766767	96.86	6.94	6	18.7
rmm5	*P. reticulata*	Male	Muscle	132590117	96.74	6.62	6	16.9
rfo1	*P. reticulata*	Female	Ovary	173872481	97.03	7.31	9	44.5
rfo2	*P. reticulata*	Female	Ovary	200875487	97.11	6.64	11	61.7
rfo3	*P. reticulata*	Female	Ovary	170461712	97.1	5.64	9	46.6
rfo4	*P. reticulata*	Female	Ovary	193904527	97.08	7.24	10	55.6
rfo5	*P. reticulata*	Female	Ovary	209981452	97.13	6.98	11	59.4
rmt1	*P. reticulata*	Male	Testis	242606019	97.13	6.92	12	69.6
rmt2	*P. reticulata*	Male	Testis	213599993	97.12	6.29	11	59.6
rmt3	*P. reticulata*	Male	Testis	177630514	97.15	5.29	9	45.2
rmt4	*P. reticulata*	Male	Testis	220792503	97.04	7.43	11	64.8
rmt5	*P. reticulata*	Male	Testis	237786687	97.16	6.76	12	64.5
wfm1	*P. wingei*	Female	Muscle	150662950	96.47	6.46	8	29.8
wfm2	*P. wingei*	Female	Muscle	131923804	96.63	7.1	6	19.3
wfm3	*P. wingei*	Female	Muscle	162035794	96.51	6.89	8	39.3
wfm4	*P. wingei*	Female	Muscle	169271566	96.45	6.86	9	41.3
wfm5	*P. wingei*	Female	Muscle	176382820	96.54	6.94	9	48.8
wmm1	*P. wingei*	Male	Muscle	168095896	96.41	7.19	8	38.9
wmm2	*P. wingei*	Male	Muscle	189950398	96.44	6.96	10	54.5
wmm3	*P. wingei*	Male	Muscle	151463025	96.4	6.13	8	33.3
wmm4	*P. wingei*	Male	Muscle	152817531	96.5	6.9	8	31.9
wmm5	*P. wingei*	Male	Muscle	213729830	96.47	6.75	11	63.7
wfo1	*P. wingei*	Female	Ovary	161547839	96.51	6.77	9	41.1
wfo2	*P. wingei*	Female	Ovary	186832493	96.45	7.38	10	54.1
wfo3	*P. wingei*	Female	Ovary	202496750	96.5	7.16	11	63.3
wfo4	*P. wingei*	Female	Ovary	148412066	96.49	7.1	8	33.4
wfo5	*P. wingei*	Female	Ovary	225556257	96.48	7.35	12	71.1
wmt2	*P. wingei*	Male	Testis	204998109	96.64	6.17	9	48.6
wmt3	*P. wingei*	Male	Testis	224531194	96.86	5.28	10	52.4
wmt4	*P. wingei*	Male	Testis	216500433	96.6	7.18	10	55.7
wmt5	*P. wingei*	Male	Testis	232179137	96.8	5.87	10	53.1

Table S2. Per-sample bisulfite conversion efficiency, estimated from genome-wide non-CpG (CHG/CHH context) methylation levels.

Sample	Non-CpG (CHG+CHH) % Methylated	Estimated Conversion Efficiency (%)
rfm1	0.615	99.385
rfm2	0.931	99.069
rfm3	0.617	99.383
rfm4	1.134	98.866
rfm5	1.325	98.675
rfo1	0.737	99.263
rfo2	0.702	99.298
rfo3	0.738	99.262
rfo4	0.749	99.251
rfo5	0.789	99.211
rmm1	0.663	99.337
rmm2	0.587	99.413
rmm3	0.701	99.299
rmm4	0.710	99.290
rmm5	0.641	99.359
rmt1	0.820	99.180
rmt2	0.783	99.217
rmt3	0.808	99.192
rmt4	0.786	99.214
rmt5	0.833	99.167
wfm1	0.657	99.343
wfm2	0.769	99.231
wfm3	0.659	99.341
wfm4	0.693	99.307
wfm5	0.664	99.336
wfo1	0.617	99.383
wfo2	0.614	99.386
wfo3	0.611	99.389
wfo4	0.647	99.353
wfo5	0.625	99.375
wmm1	0.656	99.344
wmm2	0.632	99.368
wmm3	0.637	99.363
wmm4	0.694	99.306
wmm5	0.663	99.337
wmt2	0.943	99.057
wmt3	1.014	98.986
wmt4	0.941	99.059
wmt5	1.032	98.968

Table S3. Sample-level RNA-seq sequencing and alignment quality metrics.

Sample	Species	Sex	Tissue	Total reads	% Unique mapped	% Mismatch
RO1	*P. reticulata*	Female	Ovary	47980599	96.23	0.65
RO2	*P. reticulata*	Female	Ovary	83993766	96.02	0.66
RO3	*P. reticulata*	Female	Ovary	83619993	96.14	0.62
RT1	*P. reticulata*	Male	Testis	88160403	95.64	0.66
RT2	*P. reticulata*	Male	Testis	44012212	95.92	0.67
RT3	*P. reticulata*	Male	Testis	93042726	95.72	0.66
WO1	*P. wingei*	Female	Ovary	62642648	94.29	0.96
WO2	*P. wingei*	Female	Ovary	76708600	94.58	0.93
WO3	*P. wingei*	Female	Ovary	46138971	95.65	0.77
WT1	*P. wingei*	Male	Testis	44333968	95.3	0.79
WT2	*P. wingei*	Male	Testis	161403679	95.36	0.77
WT3	*P. wingei*	Male	Testis	53078762	95.07	0.8

Table S4. GO enrichment analysis of sex-biased differentially expressed genes (DEGs).

Ont. = ontology domain (BP: biological process, CC: cellular component, MF: molecular function). DEGs in category = number of differentially methylated genes annotated to the GO term. FDR = Benjamini-Hochberg corrected p-value.

GO ID	Term	Ont.	DEGs in category	Total in category	FDR	Bias
Testis-biased hypomethylation						
GO:0060271	Cilium assembly	BP	61	91	1.57×10^{-16}	Testis-biased
GO:0036064	Ciliary basal body	CC	47	62	5.06×10^{-16}	Testis-biased
GO:0005930	Axoneme	CC	29	38	7.74×10^{-10}	Testis-biased
GO:1905515	Non-motile cilium assembly	BP	20	25	3.96×10^{-7}	Testis-biased
GO:0005813	Centrosome	CC	50	99	3.96×10^{-7}	Testis-biased
GO:0042073	Intraciliary transport	BP	13	13	6.17×10^{-7}	Testis-biased
GO:0005929	Cilium	CC	30	48	6.17×10^{-7}	Testis-biased
GO:0030992	Intraciliary transport particle B	CC	12	12	2.90×10^{-6}	Testis-biased
GO:0045505	Dynein intermediate chain binding	MF	20	28	1.07×10^{-5}	Testis-biased
GO:0031514	Motile cilium	CC	12	13	1.25×10^{-5}	Testis-biased
GO:0005814	Centriole	CC	25	40	1.78×10^{-5}	Testis-biased
GO:0030286	Dynein complex	CC	12	13	4.01×10^{-5}	Testis-biased
GO:0007018	Microtubule-based movement	BP	42	87	4.01×10^{-5}	Testis-biased
GO:0051959	Dynein light intermediate chain binding	MF	19	28	1.16×10^{-4}	Testis-biased
GO:0008569	Minus-end-directed microtubule motor activity	MF	12	14	2.29×10^{-4}	Testis-biased

GO:0035869	Ciliary transition zone	CC	13	17	2.35×10^{-4}	Testis-biased
GO:0008017	Microtubule binding	MF	73	194	4.21×10^{-4}	Testis-biased
GO:0044782	Cilium organization	BP	11	14	8.43×10^{-4}	Testis-biased
GO:0036158	Outer dynein arm assembly	BP	9	10	8.70×10^{-4}	Testis-biased
GO:0097730	Non-motile cilium	CC	9	10	9.82×10^{-4}	Testis-biased
GO:0000922	Spindle pole	CC	17	30	0.004	Testis-biased
GO:0015074	DNA integration	BP	8	10	0.005	Testis-biased
GO:0005815	Microtubule organizing center	CC	15	25	0.006	Testis-biased
GO:0003341	Cilium movement	BP	10	14	0.008	Testis-biased
GO:0030141	Secretory granule	CC	18	38	0.013	Testis-biased
GO:0061512	Protein localization to cilium	BP	8	10	0.014	Testis-biased
GO:0004252	Serine-type endopeptidase activity	MF	57	176	0.027	Testis-biased
GO:0097546	Ciliary base	CC	7	9	0.037	Testis-biased
GO:0018107	Peptidyl-threonine phosphorylation	BP	15	29	0.048	Testis-biased
GO:0000132	Establishment of mitotic spindle orientation	BP	10	16	0.048	Testis-biased
GO:0034451	Centriolar satellite	CC	9	14	0.050	Testis-biased
GO:0005313	L-glutamate transmembrane transporter activity	MF	14	27	0.050	Testis-biased
Ovary-biased hypomethylation						
GO:0005730	Nucleolus	CC	80	136	2.58×10^{-8}	Ovary-biased

GO:0030182	Neuron differentiation	BP	41	66	7.30×10^{-5}	Ovary-biased
GO:0000978	RNA polymerase II cis-regulatory region sequence-specific DNA binding	MF	327	855	1.90×10^{-4}	Ovary-biased
GO:0045165	Cell fate commitment	BP	20	31	0.015	Ovary-biased
GO:0032040	Small-subunit processome	CC	21	33	0.032	Ovary-biased
GO:0005109	Frizzled binding	MF	16	24	0.032	Ovary-biased
GO:0005125	Cytokine activity	MF	35	67	0.032	Ovary-biased
GO:0005739	Mitochondrion	CC	216	560	0.032	Ovary-biased
GO:0030687	Preribosome, large subunit precursor	CC	12	15	0.037	Ovary-biased
GO:0015267	Channel activity	MF	12	15	0.046	Ovary-biased
